# Expression and prognostic potential of PLEK2 in head and neck squamous cell carcinoma based on bioinformatics analysis

**DOI:** 10.1002/cam4.4163

**Published:** 2021-07-30

**Authors:** Jingyun Wang, Zhuang Sun, Jing Wang, Qihai Tian, Runda Huang, Hanyu Wang, Xiaohui Wang, Fei Han

**Affiliations:** ^1^ Department of Radiation Oncology Sun Yat‐sen University Cancer Center Guangzhou Guangdong Province People’s Republic of China; ^2^ State Key Laboratory of Oncology in South China Guangzhou Guangdong Province People’s Republic of China; ^3^ Collaborative Innovation Center for Cancer Medicine Guangzhou Guangdong Province People’s Republic of China; ^4^ Guangdong Key Laboratory of Nasopharyngeal Carcinoma Diagnosis and Therapy Guangzhou Guangdong Province People’s Republic of China; ^5^ West China School of Medicine Sichuan University Chengdu Sichuan Province People’s Republic of China

**Keywords:** focal adhesion, head and neck squamous cell carcinoma, ITGA3, metastasis, PLEK2

## Abstract

**Background:**

PLEK2 (pleckstrin) could bind to membrane‐bound phosphatidylinositols and further promote cell spread. Recently, several studies have noted the importance of PLEK2 in tumor metastasis. However, the role of PLEK2 in head and neck squamous cell carcinoma (HNSCC) remains to be elucidated.

**Methods:**

The PLEK2 expression in HNSCC was identified using Oncomine, Gene Expression Omnibus (GEO), UALCAN databases, and western blot analysis. Prognosis analysis was performed using Kaplan–Meier plotter, DriverDBv3, UALCAN, UCSC Xena, and GEO databases. Single‐cell functional analysis was further performed using the cancerSEA database. The PLEK2‐related co‑expressed genes were identified, and gene set enrichment analysis was performed using LinkedOmics. Furthermore, the top 10 hub genes were identified using the cytoHubba plug‐in of Cytoscape. Then, gene enrichment analysis, pathway activity, and drug sensitivity analyses of the hub genes were performed using the R package “clusterProfiler” and GSCAlite. Finally, the UCSC Xena browser was utilized to explore the hub gene most likely to play a synergic role with PLEK2 in HNSCC.

**Results:**

Elevated expression of PLEK2 was observed in HNSCC and even in HNSCC subgroups based on diverse clinicopathological features, portending a poor prognosis in HNSCC. PLEK2 was correlated with metastasis and hypoxia in HNSCC, and the PLEK2‐related co‐expressed genes were mainly involved in the focal adhesion pathway. The top 10 hub genes were primarily enriched in focal adhesion, HPV infection, ECM‐receptor interaction, and PI3K‐AKT signaling pathway, and epithelial–mesenchymal transition pathway was activated. Furthermore, the expression levels of the hub genes were associated with sensitivity and resistance to various small molecules and anti‐cancer drugs. Further study suggested that ITGA3 and PLEK2 might be viewed as inextricably linked in facilitating HNSCC metastasis.

**Conclusions:**

In general, PLEK2 might serve as a potential biomarker for the diagnosis of HNSCC and guide the development of targeted therapies for HNSCC.

## Graphical abstract

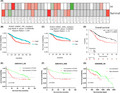

## 1 INTRODUCTION

Head and neck cancer is the seventh most common carcinoma, mainly including oral, nasopharyngeal, oropharyngeal, laryngeal, and hypopharyngeal cancers, more than 90% of which is squamous cell carcinoma.^1^ The latest data reported 931,931 new cases and 467,125 deaths of head and neck cancer in 2020 globally.^2^ Tobacco and alcohol are considered as the high‐risk factors for head and neck squamous cell carcinoma (HNSCC). In addition, many studies have indicated that human papillomavirus (HPV) infection is closely associated with oropharyngeal carcinoma.^3^ Currently, the multidisciplinary treatment, including surgery, radiotherapy, chemotherapy, immunotherapy, and targeted therapy, has achieved a favorable outcome for patients with HNSCC. However, the prognosis of advanced HNSCC patients remains unsatisfactory, attributing to the high incidence of recurrence and metastasis. Therefore, it is necessary to discover the biomarker associated with metastasis and prognosis of HNSCC.

PLEK2 (pleckstrin) is a 353 amino acid protein with a pleckstrin homology (PH) domain at each end of the molecule and a disheveled‐Egl‐10‐pleckstrin (DEP) domain in the intervening sequence.^4^ PLEK2 could bind to membrane‐bound phosphatidylinositols generated by phosphatidylinositol 3‐kinase (PI3K) and contribute to lamellipodia formation and orchestrate cytoskeletal arrangement, further inducing actin‐dependent cell spreading.^5^, ^6^ Recently, several studies have noted the importance of PLEK2 in tumor invasion and metastasis. For example, Shen et al. reported that PLEK2 could serve as a promoter of gallbladder cancer invasion and metastasis by activating EGFR/CCL2 signaling pathway.^7^ Additionally, PLEK2 could promote metastasis and vascular invasion in non‐small cell lung cancer,^8^ and its overexpression might be associated with poor progression‐free survival in patients with lung adenocarcinoma.^9^ However, limited studies have lucubrated the role of PLEK2 in tumorigenesis and progression. As we know, the research about the function of PLEK2 in HNSCC is still lacking.

In this study, we aimed to conduct a series of bioinformatics analyses to explore the pivotal role of PLEK2 in HNSCC, including comparing the expression level of PLEK2 between tumor tissues and normal tissues, exploring the effect of PLEK2 expression on HNSCC prognosis, investigating the co‐expressed genes of PLEK2 in HNSCC. Furthermore, functional enrichment analyses of PLEK2‐related co‐expressed genes were performed, and the top 10 hub genes were identified, providing further comprehension of molecular biological mechanisms in HNSCC.

## 2 MATERIALS AND METHODS

### 2.1 Samples and data collected for our study

The TCGA HNSCC data, including RNA‐sequencing data and clinical data, were downloaded from the UCSC Xena website (http://xena.ucsc.edu/).^10^

### 2.2 Gene expression analysis

The transcription level of the PLEK2 in pan‐cancers was identified in CCLE (Cancer Cell Line encyclopedia; https://portals.broadinstitute.org/ccle/data)^11^ and the "Gene_DE" module of TIMER2 (Tumor immune estimation resource, version 2; http://timer.comp‐genomics.org/).^12^ The Oncomine database (https://www.oncomine.org/resource/login.html)^13^ was used to observe the expression of PLEK2 in diverse cancers. The cutoffs of *p*‐value, fold change (FC), gene ranking was set to 0.01, 2, top 10%, respectively, and the data type was defined as mRNA.

Besides, the Oncomine platform and Gene Expression Omnibus (GEO) datasets (accession: GSE30784, GSE23558, GSE53819, GSE29330, GSE58911) were also used to collect the mRNA expression of PLEK2 in different datasets of HNSCC. The UALCAN portal (http://ualcan.path.uab.edu/analysis‐prot.html)^14^ was utilized to explore the transcription in HNSCC sub‐groups based on several clinicopathological features including gender, age, race, tumor grade, individual cancer stage, nodal metastasis status, HPV status, and TP53 status. The data collected from the UCSC Xena website was used to explore the expression of PLEK2 in HNSCC sub‐groups based on alcohol and tobacco smoking history. The HPV status in TCGA database was determined by p16 immunohistochemical (IHC) method, HPV DNA in situ hybridization (ISH) method, or using an empiric definition of >1000 mapped RNA sequencing reads (E6 and E7).^15^

### 2.3 Cell culture

Human head and neck cancer cell lines CAL27, SAS, and CAL33 were obtained from Nanjing Cobioer Biotechnology Co., Ltd. Human head and neck cancer cell line SCC25 and normal human oral keratinocyte (HOK) were donated by Professor Musheng Zeng from State Key Laboratory of Oncology in South China. All cells were cultured in Dulbecco's Modified Eagle's Medium (DMEM, Gibco) supplemented with 10% fetal bovine serum (FBS, ExCell Bio).

### 2.4 Western blot analysis

Whole‐cell lysates were extracted using RIPA lysis buffer (Beyotime) on ice for 30 min. Then the supernatants were collected by centrifugation (1400 g for 15 min at 4°C). Sample protein concentrations were quantified by protein BCA Assay (Thermo Scientific). The protein samples were electrophoresed in 12% SDS‐PAGE after boiling for 10 min, and then transferred to a PVDF membrane. The membrane was blocked in 5% bovine serum albumin (BSA) at room temperature for 2 h, and then incubated overnight with primary anti‐PLEK2 antibody (1:1000, Abclonal) and anti‐β‐tubulin antibody (1:1000, Proteintech) at 4°C. After 3 washes in TBST (10 min each), the membrane was incubated with the second antibody (1:5000, Proteintech) at room temperature for 2 h, followed by 3 washes in TBST. The protein bands were visualized using BeyoECL Plus reagent (Beyotime).

### 2.5 Survival prognosis analysis

The “Gene Summary” module of DriverDBv3 (http://ngs.ym.edu.tw/driverdb)^16^ was used to obtain the overall survival (OS) map of PLEK2 in all TCGA cancer types. The Kaplan‐Meier (KM) survival curves were plotted using KM plotter (http://kmplot.com/analysis/),^17^ DriverDBv3, UALCAN. Prognosis validation was performed using the GEO datasets (accession: GSE41613, GSE65858). The subgroup survival analysis of HNSCC patients by gender, age, tumor grade was performed by UALCAN. The subgroup survival analysis by alcohol, tobacco smoking history, TP53 mutation status, and HPV status was performed by UCSC Xena browser.

### 2.6 Genetic alteration analysis and alteration‐related prognosis

cBioPortal database was used to investigate the alteration frequency, mutation sites of PLEK2 in HNSCC and explore the prognostic difference between the altered group and unaltered group (http://www.cbioportal.org).^18^ Besides, the Catalogue of Somatic Mutations in Cancer (COSMIC) database was further employed to investigate the mutation types and substitutional mutation types of PELK2 in HNSCC.^19^

### 2.7 Single‐cell functional analysis

CancerSEA,^20^ a database designed to depict 14 functional states of 41,900 cancer cells from 25 tumor types at the single‐cell level, was used to assess what role PLEK2 might play in HNSCC.

### 2.8 Co‑expressed Gene Prediction and Gene Set Enrichment Analysis (GSEA)

PLEK2‐related co‑expressed genes were identified from the TCGA HNSCC cohort (n = 520) using the LinkFinder module of the LinkedOmics database (http://www.linkedomics.org/).^21^ For correlation analysis, the Spearman correlation test was used. Enrichment analysis was performed using the LinkInterpreter module of LinkedOmics. The tool GSEA was selected to perform the enrichment analyses, including GO analysis [biological processes (BP), cellular components (CC), and molecular functions (MF)], KEGG pathway, kinase target, and transcription factor (TF) target. False Discovery Rate (FDR) <0.05 as the rank standard, 3 minimum number of genes, and 500 simulations were performed. Protein‐protein interaction (PPI) networks of PLEK2‐related kinase target and TF target gene sets were constructed using GeneMANIA (www.genemania.org),^22^ a friendly online database facilitating the biological understanding of behind an input gene list.

### 2.9 Identification of hub genes and analysis of their functions and relationship with prognosis

The interaction between proteins downloaded from LinkedOmics could be evaluated by the STRING (The Search Tool for the Retrieval of Interacting Genes) database (http://string.embl.de/).^23^ The minimum required interaction score was set as 0.4 to construct a PPI network. Cytoscape software^24^ (version 3.8.0) was subsequently used to visualize the PPI network. Furthermore, the MCODE (Molecular Complex Detection) plug‐in was employed to identify the most significant module with a degree cutoff = 2, node score cutoff = 0.2, k‐core = 2, and max depth =100. The cytoHubba plug‐in was employed to discover the hub genes using the maximal clique centrality (MCC) method. In addition, the correlation module of TIMER2 was used to show the correlation between PLEK2 and hub genes. Enrichment analysis of these hub genes was performed using the R package “clusterProfiler.” The enrichment thresholds for statistical significance were *p* <  0.05 and *q* <  0.1(adjusted *p*‐value by Benjamini–Hochberg method). Furthermore, pathway activity and drug sensitivity analyses of hub genes were employed using GSCAlite^25^ (www.bioinfo.life.hust.edu.cn/web/GSCALite/), a platform for gene sets cancer analysis. KM survival analysis was performed by GEPIA2 (Gene Expression Profiling Interactive Analysis, version 2; http://gepia2.cancer‐pku.cn/),^26^ KM plotter, and UALCAN databases.

### 2.10 Hierarchical clustering and correlation analysis

The hierarchical clustering heatmap of PLEK2 and hub genes in the HNSCC cohort of TCGA was established using the UCSC Xena database. The scatter plot showing the Spearman correlation between PLEK2 and its hub gene was conducted by LinkedOmics.

## 3 RESULTS

### 3.1 Expression of PLEK2 in HNSCC

**FIGURE 1:**
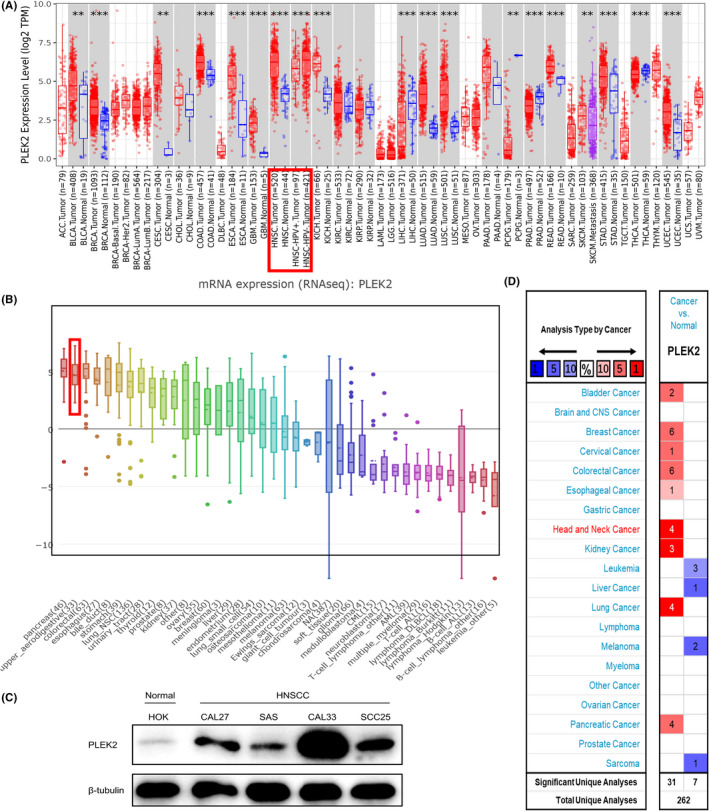
Differential expression of PLEK2 in diverse cancers. (A) The comparative expression of PLEK2 between pan‐cancers and corresponding normal tissues in the TIMER2 database. (B) mRNA expression of PLEK2 in different cancer cell lines using the CCLE database. (C) Protein expression levels of PLEK2 in normal human oral keratinocyte (HOK) and HNSCC cell lines. (D) Upregulated (red) or downregulated (blue) expression of PLEK2 across different cancers compared to normal tissues in the Oncomine database (*p < 0.05, ** *p* < 0.01, *** *p* < 0.001)

**FIGURE 2:**
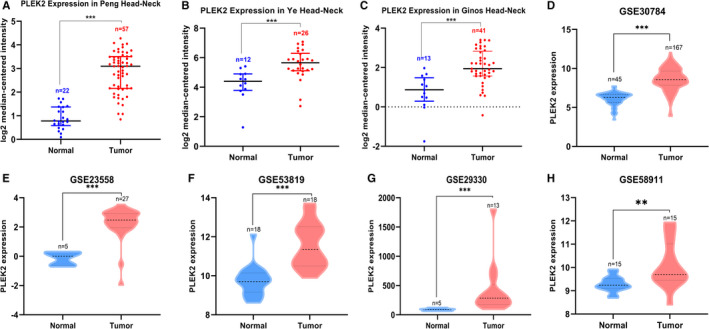
Differential expression of PLEK2 in HNSCC. (A–C) Comparison of PLEK2 expression using Oncomine database, (A) Peng‐Head Neck, (B) Ye‐Head Neck, (C) Ginos‐Head Neck. (D) Comparison of PLEK2 expression using GSE30784 dataset, (E) GSE23558 dataset, (F) GSE53819 dataset, (G) GSE29330 dataset, (H) GSE58911 dataset

To evaluate the expression difference of PLEK2 in tumors and normal tissues, the transcription levels of PLEK2 in pan‐cancers were initially analyzed using the TIMER2, CCLE, and Oncomine databases (Figure 1). Based on the data of the TIMER2 database (Figure 1A), the expression of PLEK2 was significantly upregulated in BLCA (bladder urothelial carcinoma), BRCA (breast invasive carcinoma), CESC (cervical squamous cell carcinoma and endocervical adenocarcinoma), COAD (colon adenocarcinoma), ESCA (esophageal carcinoma), GBM (glioblastoma multiforme), HNSCC, KICH (kidney Chromophobe), LUAD (lung adenocarcinoma), LUSC (lung squamous cell carcinoma), READ (rectum adenocarcinoma), STAD (stomach adenocarcinoma), UCEC (uterine corpus endometrial carcinoma) compared with normal tissues. The CCLE database revealed that the highest level of PLEK2 expression in diverse cancers was observed in cell lines of the pancreas, followed by cell lines of the upper aerodigestive tract including head and neck sites, such as mouth, pharynx, larynx (Figure 1B). Similarly, western blot results showed that HNSCC cell lines (CAL27, SAS, CAL33, SCC25) expressed higher protein expression levels of PLEK2 than normal human oral keratinocyte (HOK) (Figure 1C). Oncomine data showed that the mRNA expression levels of PLEK2 were significantly higher in HNSCC in 4 datasets (Figure 1D, Table 1, Figure S1). In the dataset of Peng Head‐Neck (Figure 2A), PLEK2 was upregulated in cancers of the oral cavity with a FC of 3.833. In Ye Head‐Neck statistics (Figure 2B), PLEK2 was higher in tongue carcinoma (FC = 2.528). Ginos Head‐Neck showed that PLEK2 was also overexpressed in HNSCC (FC = 2.450) (Figure 2C). The consistent result was achieved using the GEO dataset (GSE30784, GSE23558, GSE53819, GSE29330, GSE58911) (Figure 2D–H).

**TABLE 1:** The significant changes of PLEK2 in transcriptional level between Head and Neck Cancer and normal tissues (Oncomine Database)

Head and neck cancer		Fold change	*p* value	*t*‐test	Source/Reference
Peng Head‐Neck (79)		3.833	6.20E−20	12.782	*PLoS One* 2011/08/11
Ye Head‐Neck (38)		2.528	9.57E−4	3.561	*BMC Genomics* 2008/02/06
Ginos Head‐Neck (54)		2.450	1.91E−4	4.314	*Cancer Res* 2004/01/01
Pyeon Multi‐cancer (84)	Tongue Carcinoma vs. Normal	2.275	1.47E−5	5.440	*Cancer Res* 2007/05/15
	Oropharyngeal Carcinoma vs. Normal	2.067	0.001	4.682	*Cancer Res* 2007/05/15
	Floor of the Mouth Carcinoma vs. Normal	2.103	0.008	3.689	*Cancer Res* 2007/05/15

**FIGURE 3:**
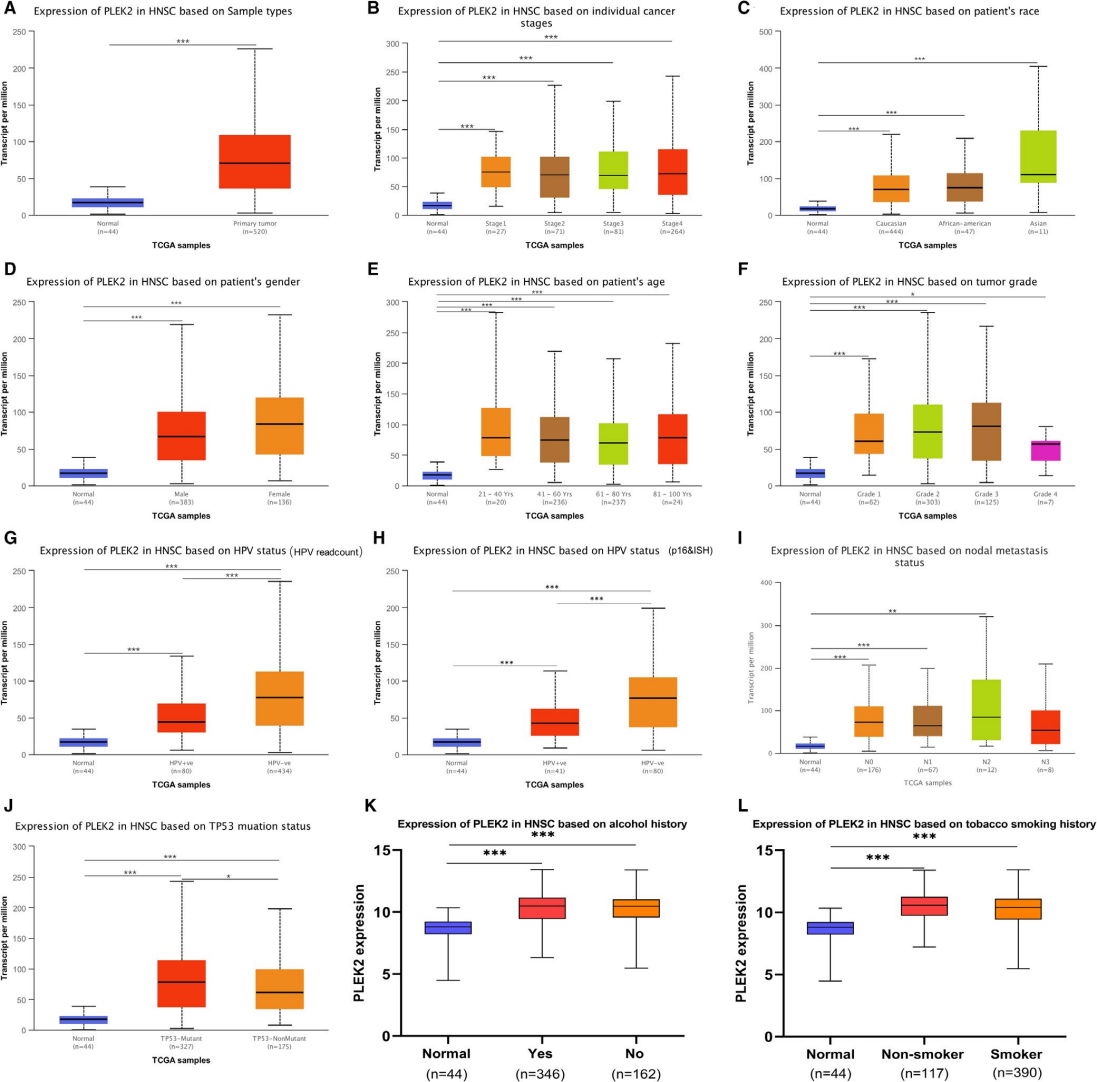
Differential expression analysis of PLEK2 in HNSCC subgroups using UALCAN and UCSC Xena database. (A) mRNA expression of PLEK2 in HNSCC and normal tissues. (B) mRNA expression of PLEK2 in HNSCC sub‐groups based on individual cancer stages, (C) race, (D) gender, (E) age, (F) tumor grade, (G) HPV status (HPV readcount), (H) HPV status (p16&ISH), (I) nodal metastasis status, (J) TP53 status, (K) alcohol history, and (L) tobacco smoking history (*p < 0.05, ** *p* < 0.01, *** *p* < 0.001)

We further evaluated the PLEK2 expression in HNSCC subgroups compared with normal samples based on several clinicopathological features including gender, age, race, tumor grade, individual cancer stages, nodal metastasis status, HPV status, and TP53 status, alcohol, and tobacco smoking history in TCGA. The clinicopathological data for HNSCC patients from TCGA was summarized in Table S1. PLEK2 was found to be upregulated in different subgroups of HNSCC (Figure 3A–L, Table S2), suggesting that PLEK2 might be a potential diagnostic marker for HNSCC patients.

### 3.2 Prognostic value of PLEK2 in HNSCC

**FIGURE 4:**
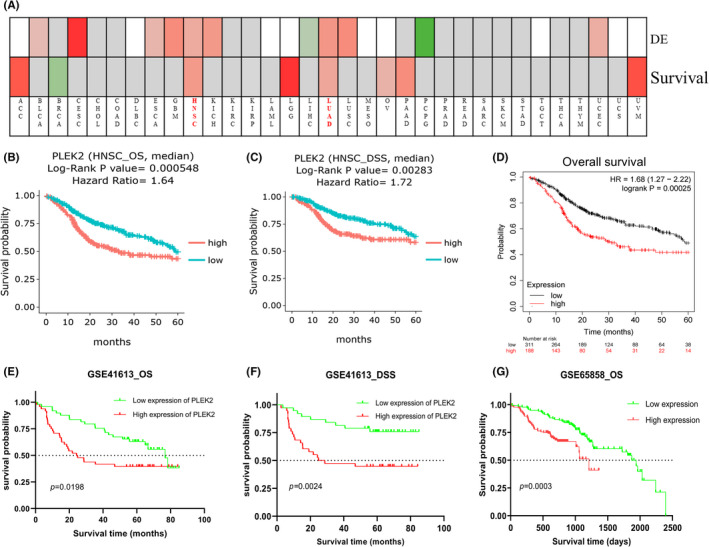
Prognostic value of PLEK2 in HNSCC. (A)The overall survival (OS) map of PLEK2 in TCGA cancers using the DriverDBv3 database. (B) KM survival curves of OS and (C) disease‐specific survival (DSS) were generated from the DriverDBv3 database. (D) KM survival curves of OS generated from KM plotter. (E) KM survival curves of OS and (F) DSS were generated from the GEO database (GSE41613). (G) KM survival curves of OS generated from the GEO database (GSE65858)

**FIGURE 5:**
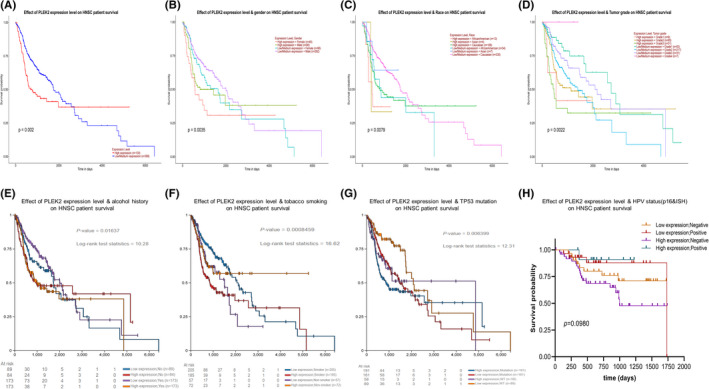
Subgroup survival analysis of HNSCC patients using UALCAN and UCSC Xena database. (A) KM survival curves of OS generated from UALCAN, (B) Subgroup survival analysis based on gender, (C) age, (D) tumor grade, (E) alcohol history, (F) tobacco smoking history, (G) TP53 mutation status, (H) HPV status

We investigated the association of PLEK2 expression and prognosis of HNSCC patients in DriverDBv3, KM plotter, UALCAN, UCSC Xena databases, and prognosis validation was performed using the GEO dataset (GSE41613, GSE65858). The OS map of PLEK2 was shown in Figure 4A. The result indicated that only HNSCC and LUAD displayed the difference both in expression and survival among all TCGA cancer types. Based on the figure of DriverDBv3 (Figure 4B–C), the high‐expression group of PLEK2 had a significantly poorer 5‐year OS [hazard ratio (HR = 1.65), *p *= 0.000548] and disease‐specific survival (DSS) (HR = 1.72, *p* = 0.00283) compared with the low‐expression group. KM plotter survival analysis showed a significant discrepancy in 5‐year OS between two groups with HR = 1.68 and *p* = 0.00025 (Figure 4D). GSE41613 dataset and GSE65858 further supported the above results (Figure 4E–G). The clinical characteristics of patients with HNSCC in GEO databases we mentioned above were all summarized in Table S3. Next, a subgroup survival analysis of HNSCC patients for OS by gender, age, tumor grade, alcohol, tobacco smoking history, and TP53 mutation status also demonstrated a significant survival difference among these subgroups (Figure 5A–G). Unfortunately, we failed to observe the survival difference among the sub‐groups based on HPV status (p16&ISH) for OS (Figure 5H, Figure S2A,B). Nevertheless, the difference in progression‐free interval (PFI) among the sub‐groups based on HPV status was statistically significant (*p* < 0.05) (Figure S2C–E). In summary, these results could highlight the potential of PLEK2 in predicting the prognosis of HNSCC.

### 3.3 The genetic alteration of PLEK2 in HNSCC and its relationship with prognosis

Increased studies have reported that the prognosis of cancers might be associated with genetic alterations. Hence, we assessed the alteration frequency of PLEK2 in 496

**FIGURE 6:**
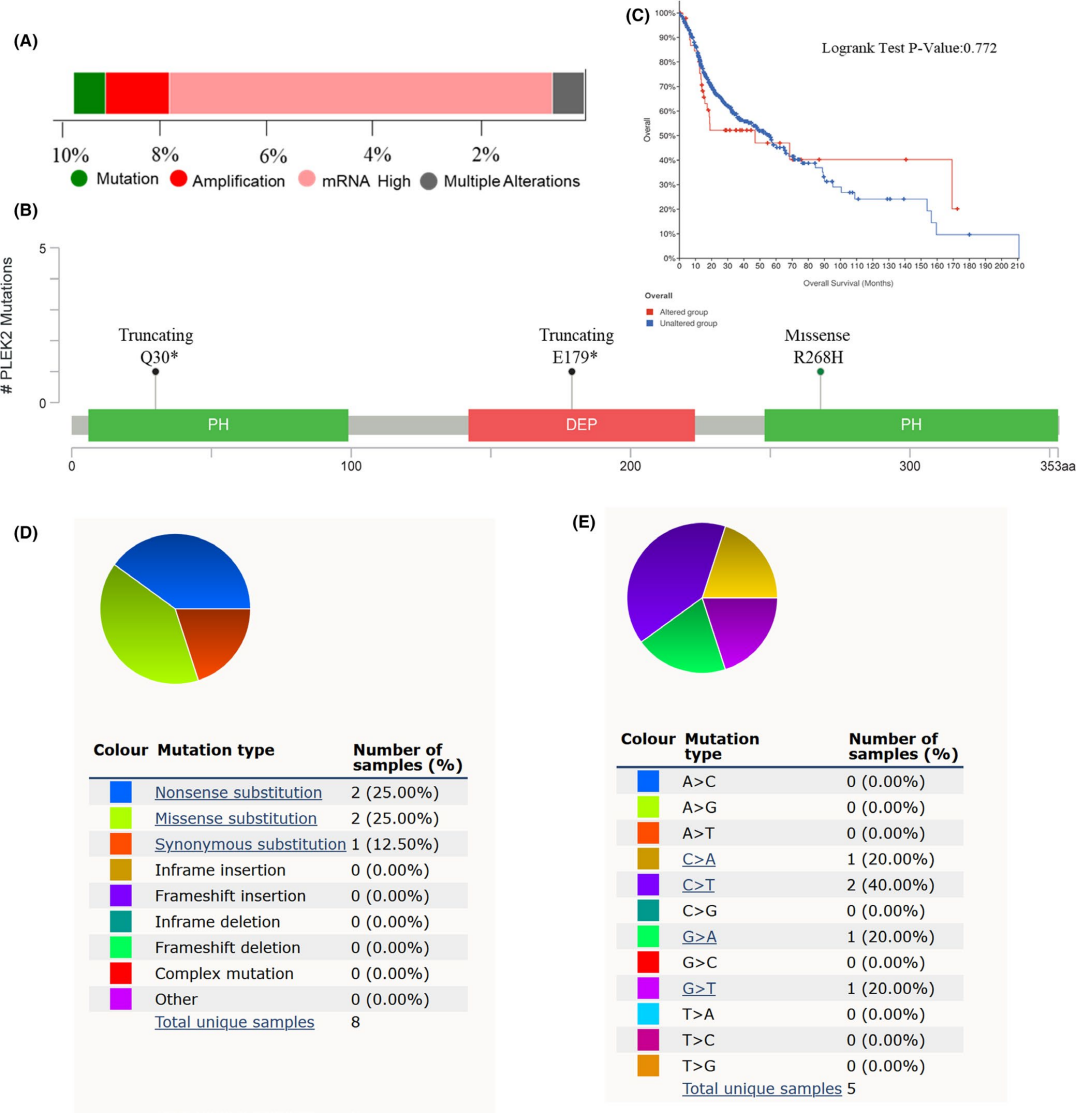
Alteration of PLEK2 in HNSCC. (A) The summary of alteration types of PLEK2 and corresponding frequencies in cBioPortal. (B) The summary of mutation sites of PLEK2 in cBioPortal. (C) The KM survival curve between PLEK2‐altered and PLEK2‐unaltered groups in cBioPortal. (D) The overview of mutation types of PLEK2 in the Catalogue of Somatic Mutations in Cancer (COSMIC). (E) The overview of substitutional mutation types of PELK2 in the COSMIC database

HNSCC cases using the cBioPortal database. As shown in Figure 6A, PLEK2 altered in 9.68% of 496 HNSCC cases. Alteration types of PLEK2 included mutation, amplification, mRNA high, and multiple alterations, with the alteration frequency of 0.6% (3/496), 1.21% (6/496), 7.26% (36/496), and 0.6% (3/496), respectively. The mutation sites of PLEK2 were further presented in Figure 6B. We found the missense mutation type occurred in PH (R268H), one of the mutation types of truncating occurred in PH(Q30*), the other occurred in DEP(E179*). No significant difference was observed in OS probability between the altered and unaltered groups, with overall median months of 46.98 and 56.44, respectively. (log‐rank test, *p* = 0.772, Figure 6C). The result indicated the adverse outcome caused by the overexpression of PLEK2 might not be due to the overall alteration of PLEK2. Next, another database, COSMIC, was further used to investigate the mutation types and substitutional mutation types of PELK2 in HNSCC. Nonsense substitutions and missense substitutions both occurred in 25% of the samples, and synonymous substitutions occurred in 12.5% of the samples (Figure 6D). The substitution mutations mainly occurred in C > T (40.00%), followed by C > A (20.00%), G > T (20.00%) and G > A (20.00%) (Figure 6E).

### 3.4 Single‐cell functional analysis

**FIGURE 7:**
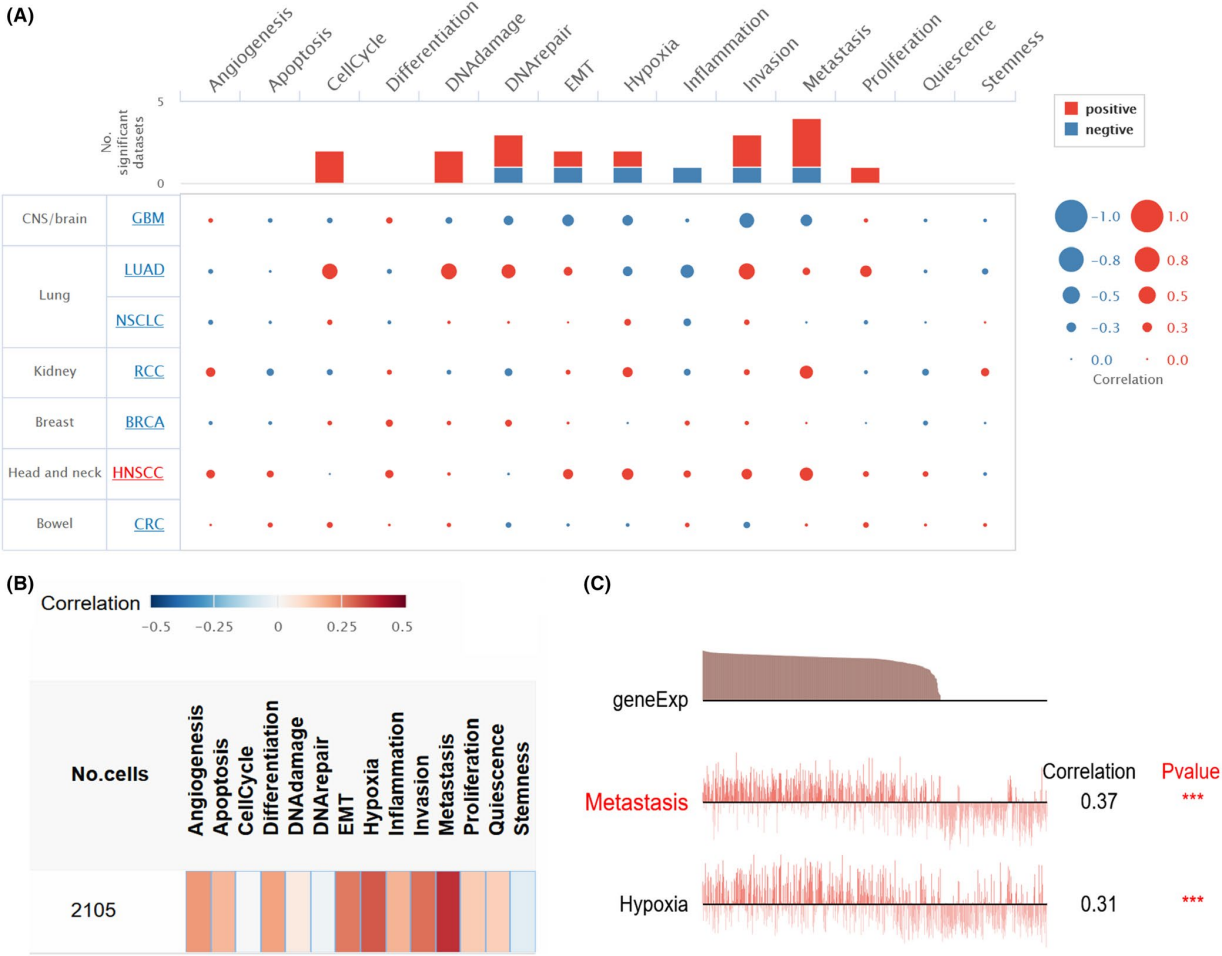
Single‐cell functional analysis of PLEK2 using CancerSEA. (A, B) The PLEK2‐related overview of functional states in HNSCC. Red and blue represent a positive correlation and a negative correlation, respectively. (A)The size of the dot and (B) the depth of the color indicates the average correlation. (C) The functional states are significantly related to PLEK2 in the EXP0063 dataset. Significance was set at *p* < 0.05

CancerSEA, a single‐cell database, was utilized to further understand the role that PLEK2 might play in single HSNCC cells. Results are summarized in Figure 7 and Figure S3). PLEK2 was found to be mainly involved in metastasis and hypoxia (Figure 7A,B). Puram SV (EXP0063) showed PLEK2 overexpression was positively correlated with metastasis and hypoxia in Figure 7C (Spearman's coefficients = 0.37, 0.31, respectively; *p* < 0.001).

### 3.5 GSEA analysis of PLEK2‑related co‑expressed genes in HNSCC

**FIGURE 8:**
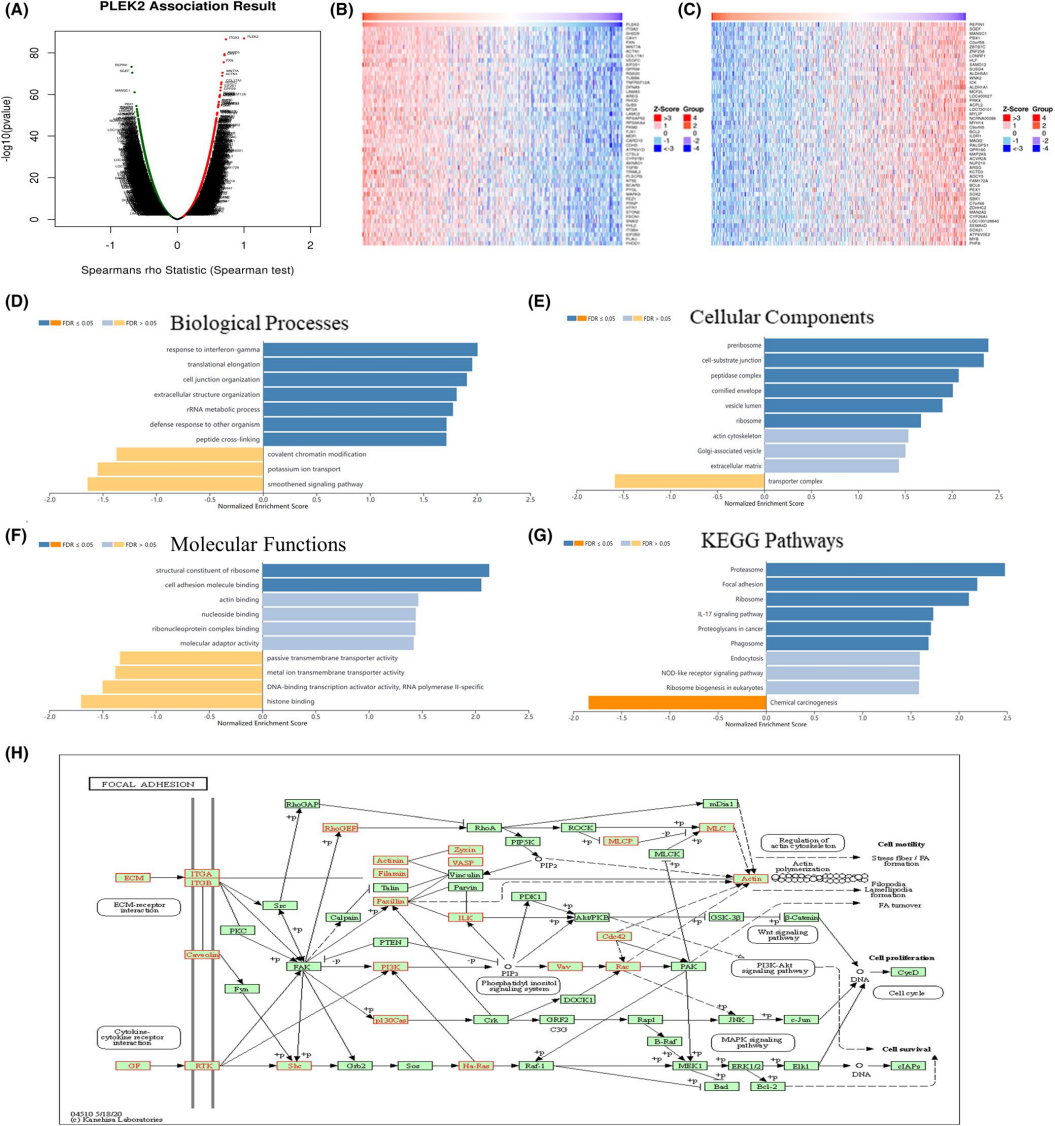
Co‑expressed gene prediction and Gene set enrichment analysis (GSEA). (A) The volcano plot representing the PLEK2‐related differentially co‐expressed genes (Spearman correlation test). The red dots representing the positively correlated genes and the green dots representing the negatively correlated genes. (B) Heat map representing the top 50 significant genes positively and (C) negatively correlated with PLEK2 in HNSCC. (D–G) The significant enrichment analysis of PLEK2 co‐expressed genes in HNSCC using GSEA. (D) Biological processes, BP. (E) Cellular components, CC. (F) Molecular functions, MF. (G) KEGG pathway. (H) KEGG pathway annotation of the focal adhesion. Red representing the leading Edge Genes of hub genes

**FIGURE 9:**
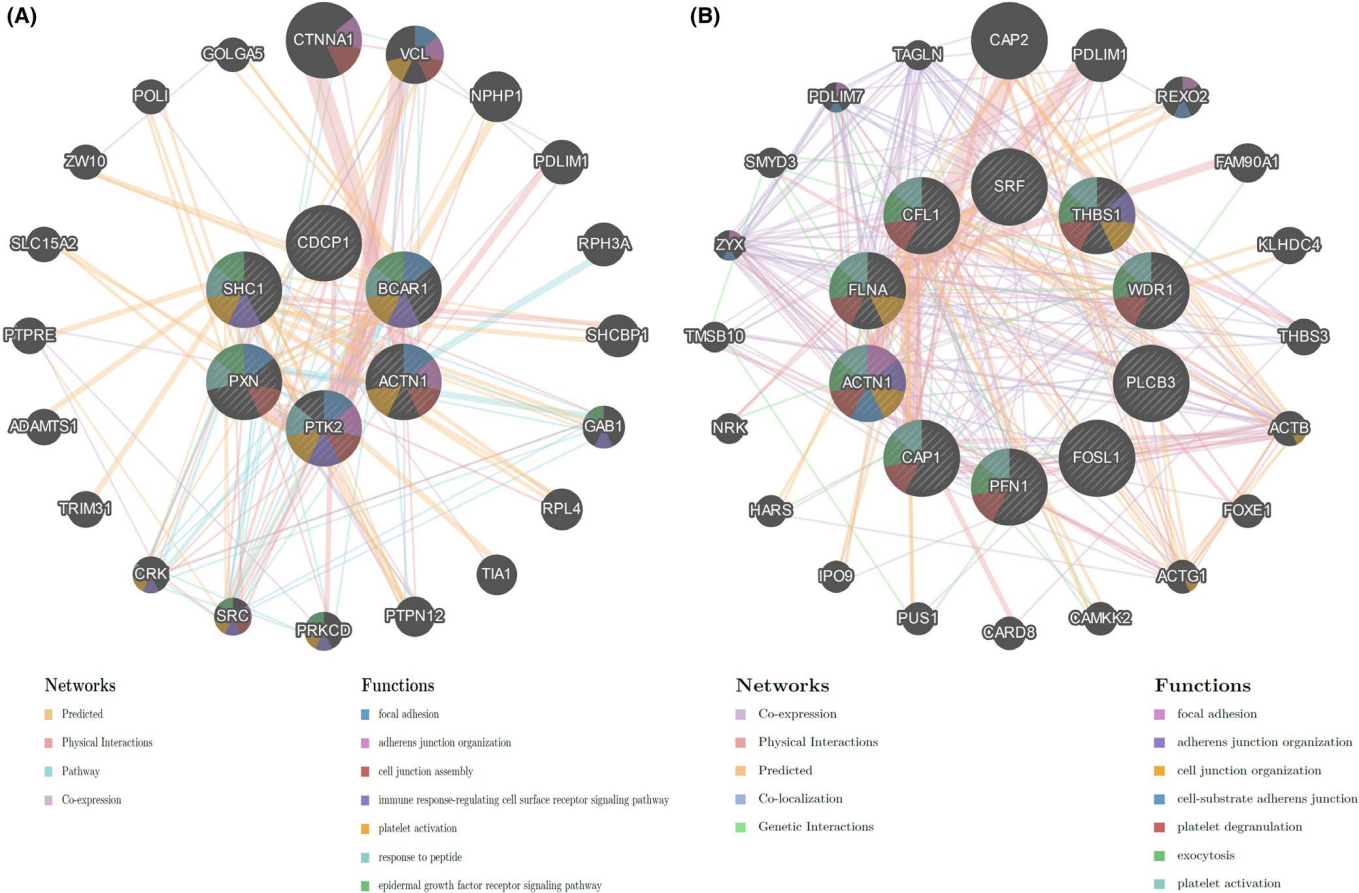
Protein‐protein interaction (PPI) network of kinase target PTK2 and transcription factor V$SRF_01 (GeneMANIA). (A) PTK2. (B) V$SRF_01. The line color of the network representing the types of interaction. The node color representing the biological functions of the gene sets

We employed the LinkedOmics database to identify the PLEK2‐related co‑expressed genes further and predicted their function in HNSCC. As indicated in the volcano plot (Figure 8A), 2970 genes were positively related with PLEK2 represented by red dots, and 6916 genes were negatively associated with PLEK2 represented by green dots [FDR(BH) < 0.001]. The heat maps exhibited the top 50 PLEK2‑related co‑expressed positive and negative genes in Figure 8B,C, and the correlation coefficients were presented in Table S4–S5. The results above indicated that PLEK2 might play a vital role in the transcriptome. GSEA tool was further conducted to identify the significant GO, KEGG pathway, kinase target, and TF target. GO results revealed that PLEK2‐related co‑expressed genes were mainly involved in cell‐substrate junction, cell junction organization, and cell adhesion molecule binding. KEGG pathway analysis indicated enrichment in focal adhesion, associated with tumor metastasis (Figure 8D–G, Table S6). The KEGG pathway annotation of the focal adhesion was presented in Figure 8H. Kinase target and TF target networks were also identified to validate the function of PLEK2 in HNSCC. The representative significant kinase target and TF target networks were protein tyrosine kinase 2 (PTK2) and TF V$SRF_01, respectively. The PPI network constructed by GeneMANIA elaborated the biological functions among genes related to Kinase_PTK2 and TF V$SRF_01. The gene sets enriched for Kinase_PTK2 and TF V$SRF_01 were both involved in focal adhesion and adherens junction organization (Figure 9A,B, Table S7). These results further validated that PLEK2 might promote HNSCC metastasis through focal adhesion.

### 3.6 Identification of hub genes and analysis of their functional and prognosis

**FIGURE 10:**
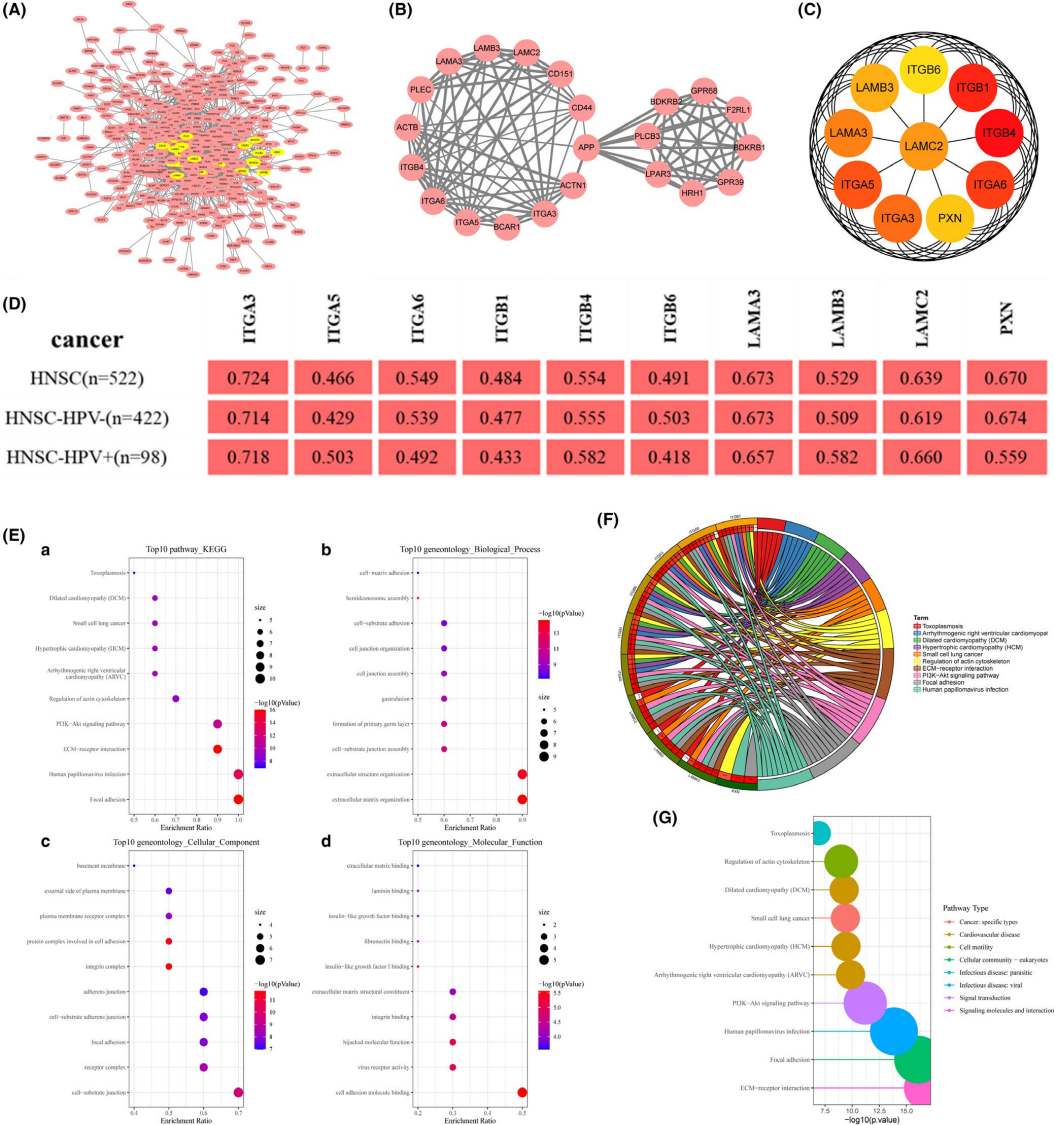
Hub genes analysis. (A) PPI network was obtained from the STRING database. (B) The PPI network of the most significant module by MCODE analysis. (C) The PPI network of the top 10 hub genes by cytoHubaa analysis. (D) The correlation coefficient between PLEK2 and the top 10 hub genes by the TIMER2 database. (E) The enrichment bubble plot of the 10 hub genes, (a) KEGG analysis, (b) BP, (c) CC, (d) MF. (F) The chord plot of KEGG pathways. (G) The classification map of pathway types

**FIGURE 11:**
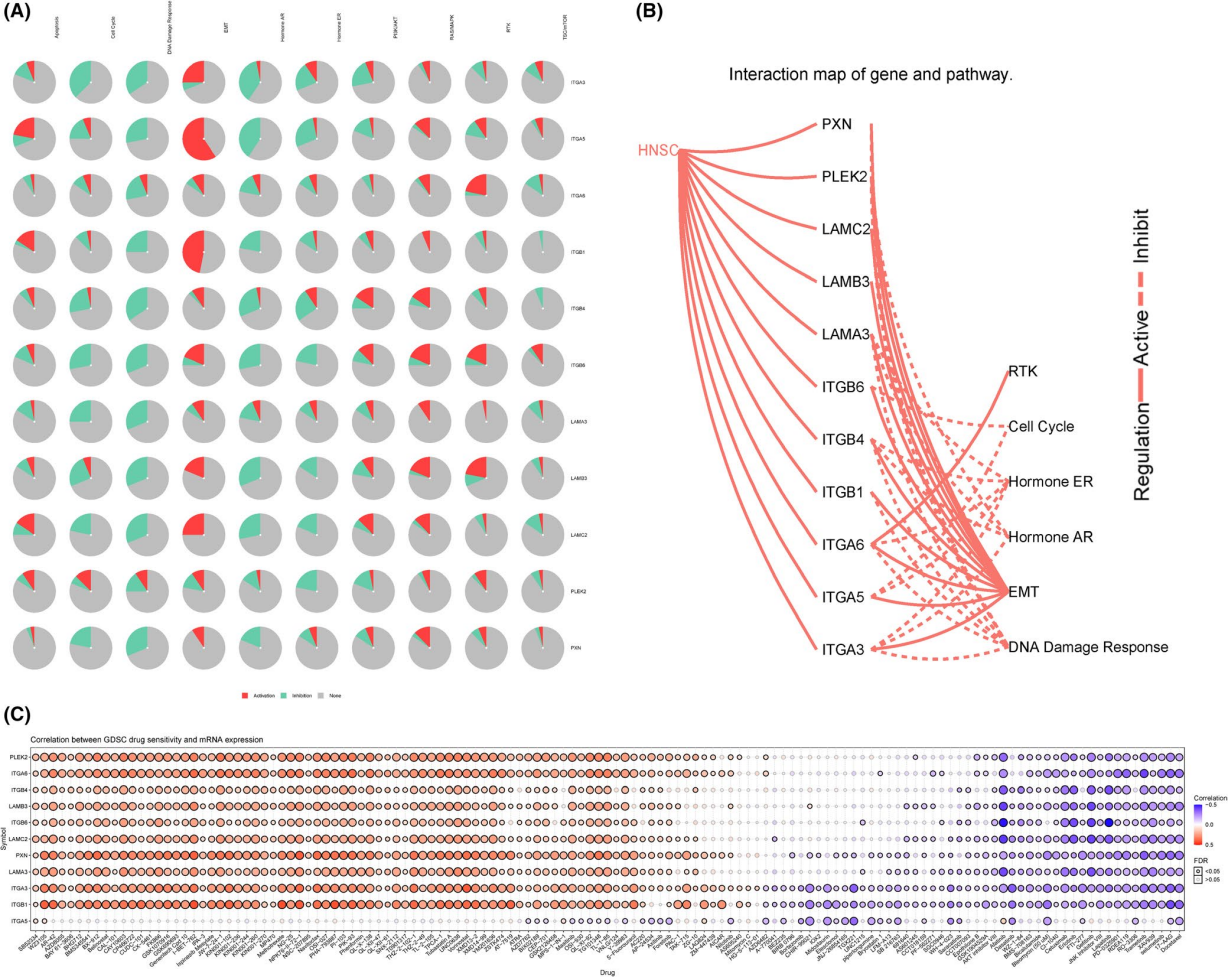
GSCAlite analysis of PLEK2 and its hub genes. (A) The plot of the global percentage of PLEK2‐related hub genes. (B) The interaction map of PLEK2‐related hub genes and pathways. (C) Drug sensitivity analysis in Genomics of Drug Sensitivity in Cancer. The Color indicated Spearman's correlation coefficient and the circle size represented the False Discovery Rate (FDR) value

The top 500 co‐expressed genes were selected to construct the PPI network by STRING database (Figure 10A). Next, the MCODE plug‐in of Cytoscape was used to identify the most crucial module (Figure 10B), and cytoHubba plug‐in was used to screen the top 10 hub genes, ITGB4, ITGB1, ITGA6, ITGA5, ITGA3, LAMA3, LAMC2, LAMB3, PXN, ITGB6 ranked by degree score (Figure 10C). The correlation coefficients between the top 10 hub genes and PLEK2 were presented using the TIMER2, GEPIA2, and cBioPortal databases (Figure 10D, Table S8). GO and KEGG analyses were summarized in Figure 10E–G and Table S9. In terms of BP, extracellular matrix organization and extracellular structure organization were most heavily enriched. As for CC, the cell‐substrate junction showed the highest enrichment. Cell adhesion molecule binding showed the highest degree of MF enrichment. The pathways with the highest enrichment were focal adhesion, HPV infection, ECM‐receptor interaction, and PI3K‐AKT signaling pathway. In addition, pathway activity and drug sensitivity analyses of hub genes were performed using GSCAlite. The pathway activity indicated that epithelial–mesenchymal transition (EMT), a vital step in tumor invasion and metastasis, was mainly activated (Figure 11A,B). We next performed the drug sensitivity analysis among PLEK2 and its hub genes in Genomics of Drug Sensitivity in Cancer (GDSC) (Figure 11C). We found that the correlation trend of gene expression levels and drug sensitivity are similar among PLEK2 and its hub genes except for ITGA5. Overexpression of these genes was associated with the sensitivity and resistance to a variety of small molecules and anti‐cancer drugs. Notedly, high expression of them mainly was positively correlated with the sensitivity of Lapatinib, Cetuximab, Afatinib, Gefitinib, Erlotinib. The results above might provide the basis for drug‐targeted therapy.

We also explored the expression pattern of hub genes in HNSCC using the UALCAN database and GEO dataset (GSE30784) (Figures S4, S5) and evaluated their prognostic value in OS using the GEPIA2, KM plotter, and UALCAN databases, summarized in Table 2 and Figures S6,S7. The top 10 hub genes all expressed significantly higher in HNSCC than normal tissues based on the UALCAN database, and all genes, except LAMA3, showed differential expression based on GSE30784. Increased expression of eight genes (ITGB4, ITGA6, ITGA5, ITGA3, LAMC2, LAMB3, PXN, ITGB6) based on KM plotter and four genes (ITGA5, ITGA3, LAMC2, PXN) based on the databases (GEPIA2, KM Plotter, UALCAN) portended a worse prognosis in OS rate.

**TABLE 2:** The *p* value representing the significance for overall survival of co‐expressed hub genes associated with PLEK2 in HNSCC (GEPIA2, KM Plotter, and UALCAN databases)

Database	ITGB4	ITGB1	ITGA6	ITGA5	ITGA3	LAMA3	LAMC2	LAMB3	PXN	ITGB6
GEPIA2	0.055	0.26	0.035*	0.0023*	0.046*	0.35	0.013*	0.011*	0.0091*	0.051
KM Plotter	0.0052*	0.074	0.0001*	0.00029*	0.00014*	0.11	0.005*	0.0016*	0.00036*	0.015*
UALCAN	0.075	0.1	0.06	0.0218*	0.0023*	0.35	0.042*	0.12	0.0017*	0.057

* *p* < 0.05 was regarded as the statistically significant difference.

### 3.7 The comprehensive analysis of ITGA3

**FIGURE 12:**
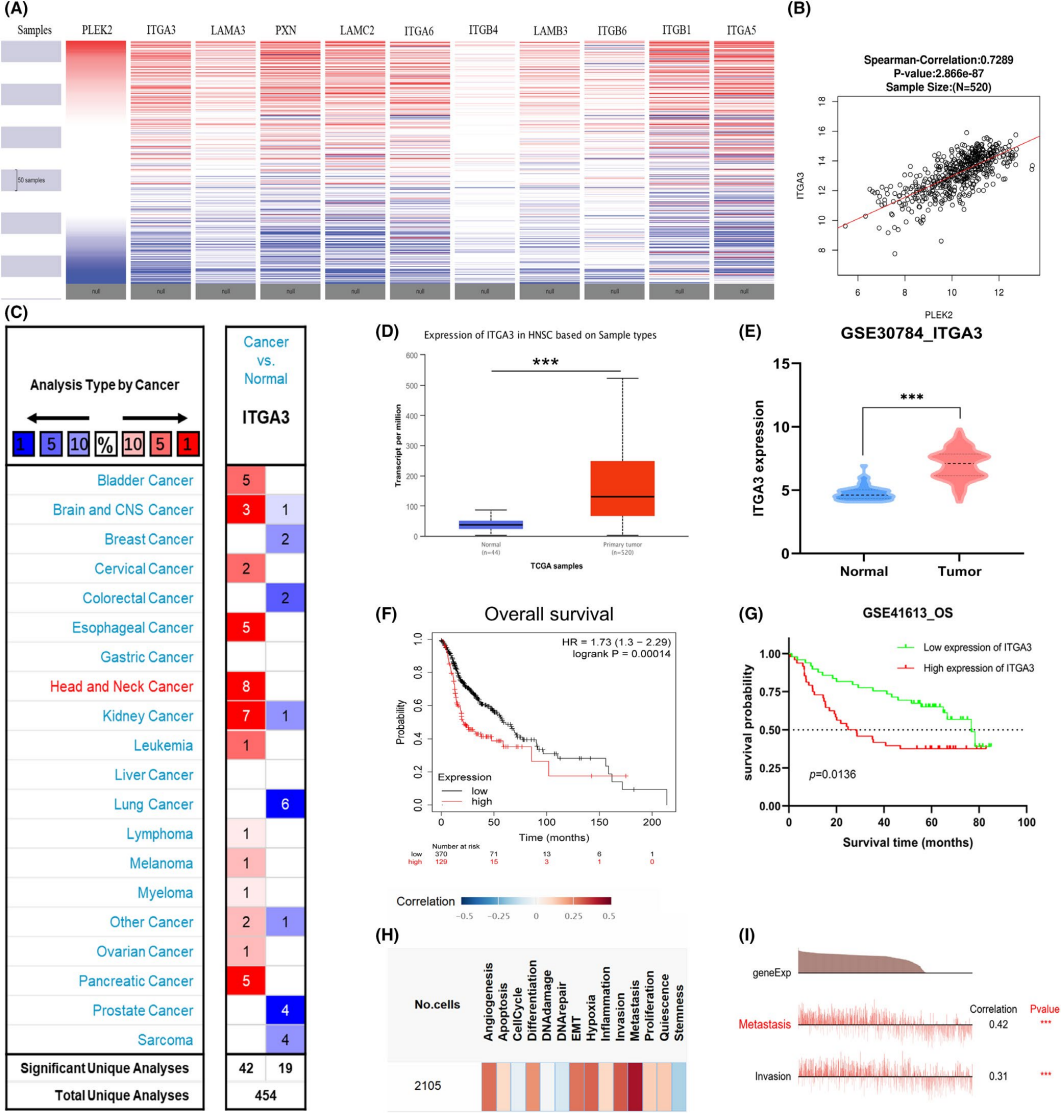
The comprehensive analysis of ITGA3. (A) The clustering heatmap for hub genes in TCGA HNSCC dataset using the UCSC Xena database. (B) The correlation coefficient between PLEK2 and ITGA3 mRNA expression in HNSCC obtained from LinkedOmics. (C) Comparison of ITGA3 expression using Oncomine database, (D) UALCAN database, (E) GEO database (GSE30784). (F) KM survival curves of OS generated from KM plotter, (G) GEO database (GSE41613). (G) The ITGA3‐related overview of functional states in HNSCC. (H) The functional states are significantly related to ITGA3 in the EXP0063 dataset

Next, we employed the UCSC Xena database to explore the target that is most likely to play a synergic role with PLEK2 in the process of pro‐metastasis. The result indicated that the expression pattern of ITGA3 was closest to that of PLEK2 (Figure 12A). Further analysis by LinkedOmics revealed that ITGA3 was highly correlated with PLEK2 in Figure 12B (Spearman's correlation = 0.7286). Subsequently, differential expression analyses were performed using data from Omcomine, TCGA, and GEO. We found that ITGA3 expressed at high levels in HNSCC tissues compared with normal tissues (Figure 12C–E). As for survival analyses, KM survival curves plotted by KM plotter and GSE41613 confirmed that high ITGA3 expression correlated with a worse prognosis (Figure 12F–G). Functional analysis performed by the CancerSEA database revealed that ITGA3 was mainly involved in the biological processes of metastasis and invasion (Figure 12H–I). In summary, ITGA3 and PLEK2 might be viewed as inextricably linked in regulating HNSCC biological characteristics.

## 4 DISCUSSION

Despite advances in treatment modalities, the mortality rate of HNSCC was as high as 50%, mainly due to loco‐regional recurrence and distant metastasis.^27^, ^28^ Therefore, it is vital to find an ideal biomarker for predicting HNSCC metastasis and prognosis. In the past few years, the association between circulating tumor cells and HNSCC metastasis and prognosis attracts growing attention.^29^, ^30^, ^31^ However, the low relative abundance of circulating tumor cells might make its detection difficult. We thus focused on finding a metastasis‐associated gene as a more sensitive biomarker for HNSCC prognosis. We analyzed the data from the TCGA database and found that PLEK2 displayed differences both in expression and survival in HNSCC and LUAD. The function and prognosis of PLEK2 in lung cancer have been reported previously. However, the role of PLEK2 in HNSCC remains to be elucidated. To our knowledge, this is the first study to comprehensively perform the expression pattern, genetic alterations, prognostic value, and functional enrichment analyses of PLEK2 in HNSCC, which might help to find novel therapeutic targets.

In our study, we first confirmed the high expression of PLEK2 in a variety of cancer tissues and cancer cell lines, including HNSCC. Furthermore, the mRNA expression profiles of PLEK2 in HNSCC were collected from multiple databases like the Oncomine, GEO, and UALCAN databases. PLEK2 was found to show upregulation in HNSCC and even in HNSCC subgroups based on clinicopathological features including gender, age, race, tumor grade, individual cancer stage, nodal metastasis status, HPV status, TP53 mutation, alcohol, and tobacco smoking history compared to normal tissues. Remarkably, subgroup analysis found that the expression level of PLEK2 in the HPV‐HNSCC group was significantly higher than HPV+HNSCC. Subgroup survival analysis revealed that the patients with the increased expression of PLEK2 and HPV+status had a better prognosis for PFI than those with high expression of PLEK2 and HPV‐ status. However, there was no significant difference in OS between the two groups. Based on the above analysis, we speculated that the HPV‐ HNSCC group with high PLEK2 expression might be considered to have a worse outcome, which is in line with many previous studies that have demonstrated that HPV+HNSCC was related to better survival prognosis.^32^, ^33^ However, considering the small number of patients with known HPV status (121/519) and the samples with high expression of PLEK2 and HPV+status (12/121) was too small, prospective research is required to further expand the sample size to confirm the results of our study. Besides, a series of survival analyses by DriverDBv3, KM plotter, UALCAN, and GSE41613 dataset indicated that high PLEK2 expression was linked to poor prognosis for patients with HNSCC. These findings suggested that PLEK2 might be considered as a potential biomarker for HNSCC. Next, we assessed the alteration frequency and mutation of PLEK2 using the cBioPortal and COSMIC databases. Unexpectedly, only 36 cases (~10%) observed PLEK2 alteration in cBioPortal, and only eight samples observed mutation in COSMIC. The alteration frequency was relatively low, and we failed to find the connection between PLEK2 alteration and HSNCC prognosis. We speculated that this result might be due to the number of samples for mutation might too small to distinguish statistical differences. Therefore, more mutation samples are required to find more meaningful results.

To further understand the underlying biological function of PLEK2 in cancer development, we searched the functional states of PLEK2 using cancerSEA and found PLEK2 was correlated with metastasis and hypoxia in HNSCC. This is the first research to investigate the role of PLEK2 in HNSCC metastasis, which is consistent with the reported pro‐metastatic function of PLEK2 in gallbladder cancer^7^ and non‐small cell lung cancer.^8^ Considering the great significance of PLEK2 in HNSCC development, we thought its expression level might affect the downstream molecules and related pathways. Therefore, the LinkedOmics database was utilized to perform PLEK2‐related neighbor gene enrichment analysis. Enrichment analysis including GO, KEGG pathway, kinase target, and TF target all indicated these PLEK2‐related genes were closely connected with cell adhesion. Hence, PLEK2 might serve as an essential core node for cell adhesion, mainly affecting the focal adhesion pathway.

More importantly, the top10 hub genes were identified, including ITGB4, ITGB1, ITGA6, ITGA5, ITGA3, LAMA3, LAMC2, LAMB3, PXN, and ITGB6. Among them, eight genes based on the KM plotter and four genes based on multiple databases had prognostic significance in HNSCC. Hub genes were subsequently subjected to functional enrichment analysis, and signaling pathways were identified, such as focal adhesion, HPV infection, ECM‐receptor interaction, and PI3K‐AKT signaling pathway. As reported, the focal adhesion pathway and ECM‐receptor interaction pathway were related to tumor progression and metastasis.^34^, ^35^, ^36^, ^37^ As aforementioned, HNSCC is HPV‐associated cancer, and hub genes were enriched in the HPV infection pathway, which might be partially be interpreted as the involvement of PLEK2‐related hub genes in the pathogenic process of HPV‐related HNSCC. The result showed that PLEK2‐related hub genes were involved in the PI3K‐AKT pathway, which was consistent with the fact that PLEK2 could bind to the membrane‐bound phosphatidylinositol produced by PI3K.^5^, ^6^ Previous studies have emphasized the importance of PI3K‐AKT in HNSCC, for example, the PI3K‐AKT signaling pathway was overactive in 90% HNSCC^38^ and was reported to mediate tumor proliferation, metabolism, inflammation, motility, and metastasis.^39^, ^40^ GSCAlite website further verified the role of PLEK2‐related hub genes in HNSCC metastasis. The pathway activity analysis further indicated that hub genes were primarily involved in EMT phenotype, a crucial step involved in tumor metastasis.^41^ Furthermore, drug sensitivity analysis revealed the sensitivity and resistance spectrum of PLEK2 and PLEK2‐related hub genes to various small molecules and other anti‐cancer drugs, which could provide support for individualized therapies and clinical decision‐making.

Integrin α3 (ITGA3), a member of the integrin alpha chain family of proteins, is a cellular adhesion molecule expressed on the cell membrane.^42^ In our study, ITGA3 had the closest expression pattern with PLEK2 in HNSCC, with a correlation coefficient of 0.73. Differential expression analysis and survival analysis demonstrated that significantly high expression of ITGA3 was observed in HNSCC versus normal tissues and correlated with a poorer outcome. Single‐cell functional analysis revealed that ITGA3 promoted HNSCC metastasis and invasion. These results were in keeping with previous observational studies.^43^, ^44^, ^45^, ^46^, ^47^ For instance, Chen et al. reported that ITGA3 might promote the progression of tongue cancer via the activation of the PI3K‐AKT signaling pathway.^43^ Nagata's research suggested that ITGA3 could act as a tumor‐promoting gene in HNSCC and be associated with an unfavorable prognosis.^44^ Moreover, another study demonstrated that the knockdown of ITGA3 could significantly inhibit HNSCC migration and invasion.^45^ The synergistic regulation of PLEK2 and ITGA3 might suggest a particular adhesion mechanism, which might facilitate HNSCC metastasis cooperatively through the cell adhesion‐related pathway.

Certainly, there were several limitations to our research that need to note. First, we cannot find an appropriate GSE dataset to simultaneously verify the differential expression and survival of PLEK2 in HNSCC and normal tissues. Second, most of our analyses were performed based on online databases. Thus, further in vitro and in vivo experiments will be required to verify our results and explore the relative mechanism. Additionally, since clinical data obtained from TCGA are incomplete, such as the high proportion of Mx in the M stage, we cannot compare the expression level of PLEK2 in the HNSCC metastatic non‐metastatic group, respectively.

## 5 CONCLUSION

Our study integrated the data obtained from public databases to gain a deeper insight into the role of PLEK2 in HNSCC. In our study, elevated expression of PLEK2 was observed in HNSCC and was found to portend a poor prognosis. PLEK2 and its co‐expressed gene ITGA3 might work in concert to promote HNSCC metastasis. In general, PLEK2 might serve as a potential biomarker for the diagnosis of HNSCC and guide the development of targeted therapies for HNSCC.

## CONFLICT OF INTEREST

The authors declare no conflicts of interest.

## AUTHORS CONTRIBUTION

Conception and design: Jingyun W, F.H; Collection and assembly of data: Jingyun W, Z.S, Jing W, X.W; Data analysis and interpretation: Q.T, R.H, H.W; Manuscript writing: Jingyun W, Z.S, Jing W; Manuscript revision: X.W, F.H; Final approval of paper: All authors.

## ETHICAL STATEMENT

The authors are accountable for all aspects of the work in ensuring that questions related to the accuracy or integrity of any part of the work are appropriately investigated and resolved.

## Supporting information

Supplementary Material

## Data Availability

The data used and/or analyzed of this study are available from the corresponding author upon reasonable request.
