# Supplementary material for: Expression and prognostic potential of PLEK2 in head and neck squamous cell carcinoma based on bioinformatics analysis
Source: Cancer Med. 2021 Jul 30;10(18):6515–33. doi: 10.1002/cam4.4163 (PMC8446404; doi:10.1002/cam4.4163)
Supplement: Supplementary file 1 — Supplementary Material [file CAM4-10-6515-s001.docx]

**Supplementary Material**

**Suppl.Table 1.** Clinical characteristics of 519 patients with HNSCC in TCGA.

|  | No. of samples | % |
| --- | --- | --- |
| Gender | | |
| Male | 383 | 73.80% |
| Female | 136 | 26.20% |
| Age at diagnosis |  |  |
| ≤60 | 257 | 49.52% |
| >60 | 262 | 50.48% |
| Clinical Stage | | |
| I | 20 | 3.85% |
| II | 97 | 18.69% |
| III | 105 | 20.23% |
| IV | 283 | 54.53% |
| Missing | 14 | 2.70% |
| Clinical T Stage | | |
| T1 | 35 | 6.74% |
| T2 | 150 | 28.90% |
| T3 | 135 | 26.01% |
| T4 | 183 | 35.26% |
| Missing | 16 | 3.08% |
| Clinical N Stage | | |
| N0 | 243 | 46.82% |
| N1 | 83 | 15.99% |
| N2 | 162 | 31.21% |
| N3 | 9 | 1.73% |
| Missing | 22 | 4.24% |
| Clinical M Stage | | |
| M0 | 488 | 94.03% |
| M1 | 6 | 1.16% |
| Missing | 25 | 4.82% |
| Pathologic Stage | | |
| I | 27 | 5.20% |
| II | 73 | 14.07% |
| III | 81 | 15.61% |
| IV | 266 | 51.25% |
| Missing | 72 | 13.87% |
| Pathologic T Stage | | |
| T0 | 1 | 0.19% |
| T1 | 48 | 9.25% |
| T2 | 135 | 26.01% |
| T3 | 99 | 19.08% |
| T4 | 174 | 33.53% |
| Missing | 62 | 11.95% |
| Pathologic N Stage | | |
| N0 | 175 | 33.72% |
| N1 | 67 | 12.91% |
| N2 | 169 | 32.56% |
| N3 | 8 | 1.54% |
| Missing | 100 | 19.27% |
| Pathologic M Stage | | |
| M0 | 185 | 35.65% |
| M1 | 1 | 0.19% |
| Missing | 333 | 64.16% |
| Histologic Grade | | |
| G1 | 62 | 11.95% |
| G2 | 303 | 58.38% |
| G3 | 125 | 24.08% |
| G4 | 7 | 1.35% |
| Missing | 22 | 4.24% |
| Alcohol History | | |
| Yes | 346 | 66.67% |
| No | 162 | 31.21% |
| Missing | 11 | 2.12% |
| Tobacco Smoking History | | |
| Non-smoker | 117 | 22.54% |
| Smoker | 390 | 75.14% |
| Missing | 12 | 2.31% |
| HPV Status by p16 Testing | | |
| Positive | 38 | 7.32% |
| Negative | 73 | 14.07% |
| Missing | 408 | 78.61% |
| HPV Status by ISH Testing | | |
| Positive | 21 | 4.05% |
| Negative | 65 | 12.52% |
| Missing | 433 | 83.43% |
| HPV Status by p16&ISH Testing | | |
| Positive | 41 | 7.90% |
| Negative | 80 | 15.41% |
| Missing | 398 | 76.69% |
| TP53 Mutation Status | | |
| Wild Type | 147 | 28.32% |
| Mutation | 352 | 67.82% |
| Missing | 20 | 3.85% |

**Suppl.Table 2.** The PLEK2 mRNA expression for HNSCC based on different clinicopalthoogical parameters using UALCAN.

| **Variables** | **Different stages** | **N** | **Comparisons** | **Statistical significance** |
| --- | --- | --- | --- | --- |
| Sample types | Normal | 44 | Normal vs. primary tumor | <1E-12 |
|  | Primary tumor | 522 |  |  |
| Individual cancer stages | Stage 1 | 27 | Normal vs. Stage 1 | 3.82920000041587E-07 |
|  | Stage 2 | 71 | Normal vs. Stage 2 | 1.04609998441418E-09 |
|  | Stage 3 | 81 | Normal vs. Stage 3 | 1.66533453693773E-15 |
|  | Stage 4 | 264 | Normal vs. Stage 4 | 1.62447832963153E-12 |
| Patients’ race | Caucasian | 444 | Normal vs. Caucasian | <1E-12 |
|  | African-American | 47 | Norma vs. AfricanAmerican | 2.02480000544014E-08 |
|  | Asian | 11 | Normal vs. Asian | 3.996700E-03 |
| Patents’  gender | Male | 383 | Normal vs. Male | <1E-12 |
|  | Female | 136 | Normal vs. Female | <1E-12 |
| Patients’ age | 21-40 yrs | 20 | Normal vs. 21-40 yrs | 3.231400E-04 |
|  | 41-60 yrs | 236 | Normal vs. 41-60 yrs | 1.62447832963153E-12 |
|  | 61-80 yrs | 237 | Normal vs. 61-80 yrs | <1E-12 |
|  | 81-100 yrs | 24 | Normal vs. 81-100 yrs | 7.92540000000841E-05 |
| Tumor grade | Grade 1-Well differentiated (low grade) | 62 | Normal vs. Grade 1 | 1.62647673107585E-12 |
|  | Grade 2- Moderately differentiated (intermediate grade) | 303 | Normal vs. Grade 2 | 1.62447832963153E-12 |
|  | Grade 3- Poorly differentiated (high grade) | 125 | Normal vs. Grade 3 | 1.62447832963153E-12 |
|  | Grade 4- Undifferentiated (high grade) | 7 | Normal vs. Grade 4 | 1.239790E-02 |
| HPV status (p16&ISH) | HPV+ve | 41 | Normal vs. HPV+ve | 7.35170000432106E-08 |
|  | HPV-ve | 80 | Normal vs. HPV-ve | 1.62447832963153E-12 |
|  |  |  | HPV+ve vs. HPV-ve | 4.805100E-04 |
| HPV status (readcount) | HPV+ve | 80 | Normal vs. HPV+ve | 1.19904086659517E-14 |
|  | HPV-ve | 434 | Normal vs. HPV-ve | <1E-12 |
|  |  |  | HPV+ve vs. HPV-ve | 1.14275255924667E-12 |
| Nodal metastasis status | No regional lymph node metastasis | 176 | Normal vs. N0 | 1.62447832963153E-12 |
|  | N1-Metastases in 1 to 3 | 67 | Normal vs. N1 | 1.70308211977499E-13 |
|  | N2-Metastases in 4 to 9 axillary lymph nodes | 12 | Normal vs. N2 | 6.717300E-03 |
|  | N3-Metastases in 10 or more axillary lymph | 8 | Normal vs. N3 | 5.653500E-02 |
| TP53  mutation status | TP53 mutant | 327 | Norma vs. TP53-Mutant | 1.11022302462516E-16 |
|  | TP53 non- mutant | 175 | Normal vs. TP53-NonMutant | <1E-12 |
|  |  |  | TP53-Mutant vs. TP53-NonMutant | 1.017600E-02 |
|  |  |  |  |  |

**Suppl.Table 3.** Clinical characteristics of patients with HNSCC in GEO databases.

|  |  | GSE30784 | GSE23558 | GSE53819 | GSE29330 | GSE58911 | GSE41613 | GSE65858 |
| --- | --- | --- | --- | --- | --- | --- | --- | --- |
| Number of samples | Tumor tissues | 167 | 27 | 18 | 13 | 15 | 97 | 270 |
|  | Normal tissues | 45 | 5 | 18 | 5 | 15 | 0 | 0 |
| Source | | Oral tissue | Oral tissue | Nasopharyngeal carcinoma | Larnyx | Larnyx | Oral squamous cell carcinoma | Larynx |
|  |  |  |  |  | Oral cavity | Hypopharynx |  | Hypopharynx |
|  |  |  |  |  | Oropharynx | Oropharynx |  | Oropharynx |
|  |  |  |  |  |  |  |  | Cavum Oris |
| Age | <60 | 90 | 20 | 13 | - | 7 | 22 | 153 |
|  | ≥60 | 77 | 7 | 5 | - | 8 | 75 | 117 |
| Gender | Male | 120 | 20 | 12 | 9 | - | 66 | 223 |
|  | Female | 47 | 7 | 6 | 3 | - | 31 | 47 |
|  | Missing | 0 | 0 | 0 | 1 | - | 0 | 0 |
| Stage | I-II | - | 3 | - | - | - | 41 | 55 |
|  | III-IV | - | 24 | - | - | - | 56 | 215 |
| T stage | I-II | - | - | - | 4 | - | - | 115 |
|  | III-IV | - | - | - | 4 | - | - | 155 |
|  | Missing | - | - | - | 5 | - | - | 0 |
| N stage | 0 | - | - | - | 5 | - | - | 94 |
|  | I-III | - | - | - | 3 | - | - | 176 |
|  | Missing | - | - | - | 5 | - |  | 0 |
| M stage | 0 | - | - | - | 8 | - | - | 263 |
|  | 1 | - | - | - | 0 | - | - | 7 |
|  | Missing | - | - | - | 5 | - | - | 0 |
| HPV status | Positive | - | - | - |  | 4 | 97 | 73 |
|  | Negative | - | - | - |  | 10 | 0 | 196 |
|  | Missing | - | - | - |  | 1 | 0 | 1 |
| Smoking history | Yes | - | - | - | 6 | - | - | 222 |
|  | No | - | - | - | 3 | - | - | 48 |
|  | Missing | - | - | - | 4 | - | - | 0 |

**Suppl.Table 4.** The correlation of the top 50 co-expressed gene positively associated with PLEK2 in HNSCC using LinkedOmics.

| Rank | Gene | Statistic | P-value | FDR (BH) | Event_SD | Event_TD |
| --- | --- | --- | --- | --- | --- | --- |
| 1 | PLEK2 | 1 | 1.00E-87 | 1.00E-83 | 520 | 520 |
| 2 | ITGA3 | 0.728862 | 2.87E-87 | 2.89E-83 | 520 | 520 |
| 3 | SH2D5 | 0.707513 | 3.96E-80 | 2.66E-76 | 520 | 520 |
| 4 | CAV1 | 0.705718 | 1.48E-79 | 7.44E-76 | 520 | 520 |
| 5 | PXN | 0.69493 | 3.25E-76 | 1.31E-72 | 520 | 520 |
| 6 | WNT7A | 0.6776 | 3.81E-71 | 9.61E-68 | 520 | 511 |
| 7 | ACTN1 | 0.672349 | 1.12E-69 | 2.51E-66 | 520 | 520 |
| 8 | COL17A1 | 0.660747 | 1.54E-66 | 3.11E-63 | 520 | 520 |
| 9 | VEGFC | 0.657126 | 1.38E-65 | 2.52E-62 | 520 | 519 |
| 10 | EIF2S1 | 0.651486 | 3.93E-64 | 6.60E-61 | 520 | 520 |
| 11 | GPR39 | 0.645964 | 9.76E-63 | 1.51E-59 | 520 | 519 |
| 12 | RGS20 | 0.63739 | 1.26E-60 | 1.69E-57 | 520 | 520 |
| 13 | TUBB6 | 0.635635 | 3.33E-60 | 4.20E-57 | 520 | 520 |
| 14 | TNFRSF12A | 0.634735 | 5.49E-60 | 6.51E-57 | 520 | 520 |
| 15 | DFNA5 | 0.634346 | 6.80E-60 | 7.61E-57 | 520 | 520 |
| 16 | LAMA3 | 0.634076 | 7.89E-60 | 8.37E-57 | 520 | 520 |
| 17 | AREG | 0.632737 | 1.65E-59 | 1.66E-56 | 520 | 520 |
| 18 | RHOD | 0.622969 | 3.17E-57 | 3.04E-54 | 520 | 520 |
| 19 | GJB3 | 0.622277 | 4.57E-57 | 4.18E-54 | 520 | 520 |
| 20 | MT2A | 0.61766 | 5.11E-56 | 4.48E-53 | 520 | 520 |
| 21 | LAMC2 | 0.616375 | 9.95E-56 | 8.36E-53 | 520 | 520 |
| 22 | RPSAP52 | 0.616123 | 1.13E-55 | 9.14E-53 | 520 | 510 |
| 23 | RPS6KA4 | 0.615177 | 1.84E-55 | 1.43E-52 | 520 | 520 |
| 24 | PKM2 | 0.612273 | 8.15E-55 | 5.87E-52 | 520 | 520 |
| 25 | FJX1 | 0.611313 | 1.33E-54 | 9.23E-52 | 520 | 520 |
| 26 | MDFI | 0.599224 | 5.37E-52 | 3.25E-49 | 520 | 520 |
| 27 | CARD10 | 0.598625 | 7.18E-52 | 4.13E-49 | 520 | 520 |
| 28 | CDH3 | 0.598042 | 9.52E-52 | 5.33E-49 | 520 | 520 |
| 29 | ATP6V1D | 0.596707 | 1.81E-51 | 9.88E-49 | 520 | 520 |
| 30 | CTSL2 | 0.596553 | 1.95E-51 | 1.04E-48 | 520 | 520 |
| 31 | CYP27B1 | 0.593967 | 6.74E-51 | 3.40E-48 | 520 | 520 |
| 32 | AKNAD1 | 0.586761 | 2.01E-49 | 9.21E-47 | 520 | 471 |
| 33 | TGFBI | 0.585942 | 2.94E-49 | 1.32E-46 | 520 | 520 |
| 34 | TRIML2 | 0.585507 | 3.60E-49 | 1.58E-46 | 520 | 436 |
| 35 | PLSCR3 | 0.583112 | 1.09E-48 | 4.47E-46 | 520 | 520 |
| 36 | NT5E | 0.580978 | 2.88E-48 | 1.08E-45 | 520 | 520 |
| 37 | BCAR3 | 0.580234 | 4.05E-48 | 1.48E-45 | 520 | 520 |
| 38 | PYGL | 0.580107 | 4.29E-48 | 1.54E-45 | 520 | 520 |
| 39 | MARK3 | 0.578538 | 8.73E-48 | 2.98E-45 | 520 | 520 |
| 40 | FEZ1 | 0.5782 | 1.02E-47 | 3.42E-45 | 520 | 520 |
| 41 | PRNP | 0.577118 | 1.66E-47 | 5.48E-45 | 520 | 520 |
| 42 | HTR7 | 0.576725 | 1.98E-47 | 6.43E-45 | 520 | 520 |
| 43 | STON2 | 0.576675 | 2.02E-47 | 6.43E-45 | 520 | 520 |
| 44 | FSCN1 | 0.576623 | 2.07E-47 | 6.43E-45 | 520 | 520 |
| 45 | SNAI2 | 0.57565 | 3.20E-47 | 9.73E-45 | 520 | 520 |
| 46 | FHL2 | 0.574989 | 4.30E-47 | 1.28E-44 | 520 | 520 |
| 47 | ITGB4 | 0.574265 | 5.94E-47 | 1.74E-44 | 520 | 520 |
| 48 | EIF2B2 | 0.57394 | 6.87E-47 | 1.98E-44 | 520 | 520 |
| 49 | PLAU | 0.572557 | 1.27E-46 | 3.51E-44 | 520 | 520 |
| 50 | FHOD1 | 0.5725 | 1.30E-46 | 3.55E-44 | 520 | 520 |

**Suppl.Table 5.** The correlation of the top 50 co-expressed gene negatively associated with PLEK2 in HNSCC using LinkedOmics.

| Rank | Gene | Statistic | | P-value | FDR (BH) | Event_SD | | Event_TD | |
| --- | --- | --- | --- | --- | --- | --- | --- | --- | --- |
| 1 | REPIN1 | | -0.68754 | 5.23E-74 | 1.76E-70 | 520 | 520 | |  |
| 2 | SGEF | | -0.67763 | 3.74E-71 | 9.61E-68 | 520 | 518 | |  |
| 3 | MANSC1 | | -0.64226 | 8.09E-62 | 1.17E-58 | 520 | 519 | |  |
| 4 | PBX1 | | -0.61425 | 2.97E-55 | 2.22E-52 | 520 | 520 | |  |
| 5 | C3orf58 | | -0.60746 | 9.23E-54 | 6.21E-51 | 520 | 520 | |  |
| 6 | ZBTB7C | | -0.60689 | 1.23E-53 | 8.01E-51 | 520 | 520 | |  |
| 7 | ZNF238 | | -0.60257 | 1.04E-52 | 6.58E-50 | 520 | 520 | |  |
| 8 | LONRF1 | | -0.59918 | 5.49E-52 | 3.25E-49 | 520 | 520 | |  |
| 9 | HLF | | -0.59535 | 3.47E-51 | 1.80E-48 | 520 | 520 | |  |
| 10 | SAMD12 | | -0.59271 | 1.23E-50 | 6.04E-48 | 520 | 519 | |  |
| 11 | SUSD4 | | -0.59163 | 2.05E-50 | 9.83E-48 | 520 | 520 | |  |
| 12 | ALDH5A1 | | -0.59138 | 2.30E-50 | 1.08E-47 | 520 | 520 | |  |
| 13 | WNK2 | | -0.58513 | 4.28E-49 | 1.83E-46 | 520 | 515 | |  |
| 14 | ICK | | -0.58403 | 7.11E-49 | 2.99E-46 | 520 | 520 | |  |
| 15 | ALDH1A1 | | -0.58264 | 1.35E-48 | 5.45E-46 | 520 | 520 | |  |
| 16 | MCF2L | | -0.58179 | 1.99E-48 | 7.86E-46 | 520 | 520 | |  |
| 17 | LOC400027 | | -0.58125 | 2.54E-48 | 9.85E-46 | 520 | 520 | |  |
| ·18 | PRKX | | -0.58105 | 2.79E-48 | 1.06E-45 | 520 | 520 | |  |
| 19 | ACPL2 | | -0.5799 | 4.72E-48 | 1.67E-45 | 520 | 520 | |  |
| 20 | LOC730101 | | -0.57859 | 8.53E-48 | 2.97E-45 | 520 | 518 | |  |
| 21 | MYLIP | | -0.57662 | 2.07E-47 | 6.43E-45 | 520 | 520 | |  |
| 22 | NCRNA00086 | | -0.57563 | 3.23E-47 | 9.73E-45 | 520 | 518 | |  |
| 23 | MYH14 | | -0.5736 | 7.99E-47 | 2.27E-44 | 520 | 520 | |  |
| 24 | C9orf45 | | -0.5728 | 1.14E-46 | 3.19E-44 | 520 | 520 | |  |
| 25 | BCL2 | | -0.57122 | 2.30E-46 | 6.09E-44 | 520 | 520 | |  |
| 26 | ILDR1 | | -0.57064 | 2.96E-46 | 7.76E-44 | 520 | 519 | |  |
| 27 | MAGI2 | | -0.56863 | 7.15E-46 | 1.82E-43 | 520 | 520 | |  |
| 28 | RALGPS1 | | -0.56854 | 7.42E-46 | 1.87E-43 | 520 | 520 | |  |
| 29 | GPR160 | | -0.56774 | 1.05E-45 | 2.62E-43 | 520 | 520 | |  |
| 30 | MAP2K6 | | -0.56743 | 1.21E-45 | 2.97E-43 | 520 | 519 | |  |
| 31 | ACVR2A | | -0.56636 | 1.92E-45 | 4.66E-43 | 520 | 520 | |  |
| 32 | NUP210 | | -0.56593 | 2.31E-45 | 5.55E-43 | 520 | 520 | |  |
| 33 | ARSG | | -0.56313 | 7.72E-45 | 1.71E-42 | 520 | 520 | |  |
| 34 | KCTD3 | | -0.56238 | 1.06E-44 | 2.28E-42 | 520 | 520 | |  |
| 35 | ADCY5 | | -0.56068 | 2.20E-44 | 4.62E-42 | 520 | 506 | |  |
| 36 | FAM172A | | -0.5606 | 2.27E-44 | 4.73E-42 | 520 | 520 | |  |
| 37 | BCL6 | | -0.55876 | 4.98E-44 | 1.01E-41 | 520 | 520 | |  |
| 38 | PEX1 | | -0.55855 | 5.42E-44 | 1.09E-41 | 520 | 520 | |  |
| 39 | SOX2 | | -0.55807 | 6.64E-44 | 1.30E-41 | 520 | 520 | |  |
| 40 | SBK1 | | -0.55747 | 8.57E-44 | 1.66E-41 | 520 | 520 | |  |
| 41 | C7orf46 | | -0.55653 | 1.27E-43 | 2.40E-41 | 520 | 518 | |  |
| 42 | ZDHHC2 | | -0.55421 | 3.35E-43 | 6.14E-41 | 520 | 520 | |  |
| 43 | MAN2A2 | | -0.55367 | 4.20E-43 | 7.49E-41 | 520 | 520 | |  |
| 44 | CYP26A1 | | -0.55367 | 4.20E-43 | 7.49E-41 | 520 | 432 | |  |
| 45 | LOC100128640 | | -0.5521 | 8.05E-43 | 1.40E-40 | 520 | 516 | |  |
| 46 | SEMA4D | | -0.55145 | 1.05E-42 | 1.81E-40 | 520 | 520 | |  |
| 47 | SOX21 | | -0.55131 | 1.11E-42 | 1.90E-40 | 520 | 515 | |  |
| 48 | ATP6V0E2 | | -0.54966 | 2.19E-42 | 3.71E-40 | 520 | 520 | |  |
| 49 | MYB | | -0.54924 | 2.60E-42 | 4.37E-40 | 520 | 520 | |  |
| 50 | PHF8 | | -0.54907 | 2.79E-42 | 4.66E-40 | 520 | 520 | |  |

**Suppl.Table 6.** The top 10 terms of gene enrichment analyses of PLEK2-related co-expressed genes by GSEA tool.

| **Gene Set** | **Description** | **Size** | **Leading Edge Number** | **ES** | **NES** | **P Value** | **FDR** |
| --- | --- | --- | --- | --- | --- | --- | --- |
| **GO: Biological Process** | | | | | | | |
| GO:0018149 | peptide cross-linking | 58 | 28 | 0.55222 | 1.7154 | 0 | 0.035801 |
| GO:0006414 | translational elongation | 123 | 54 | 0.56384 | 1.9571 | 0 | 0.0086888 |
| GO:0007224 | smoothened signaling pathway | 125 | 54 | -0.54522 | -1.6420 | 0 | 0.17997 |
| GO:0034341 | response to interferon-gamma | 187 | 46 | 0.52664 | 2.0070 | 0 | 0.0060339 |
| GO:0016072 | rRNA metabolic process | 210 | 79 | 0.47390 | 1.7771 | 0 | 0.025041 |
| GO:0006813 | potassium ion transport | 222 | 64 | -0.48527 | -1.5481 | 0 | 0.52022 |
| GO:0034330 | cell junction organization | 271 | 51 | 0.49764 | 1.9069 | 0 | 0.012413 |
| GO:0043062 | extracellular structure organization | 383 | 73 | 0.45296 | 1.8113 | 0 | 0.024538 |
| GO:0016569 | covalent chromatin modification | 424 | 97 | -0.41774 | -1.3711 | 0.0021277 | 0.49661 |
| GO:0098542 | defense response to other organism | 428 | 123 | 0.44965 | 1.7168 | 0 | 0.037358 |
| **GO: Cellular Component** | | | | | | | |
| GO:0030684 | preribosome | 66 | 36 | 0.75112 | 2.3878 | 0 | 0 |
| GO:0030055 | cell-substrate junction | 395 | 93 | 0.61087 | 2.3388 | 0 | 0 |
| GO:1905368 | peptidase complex | 85 | 42 | 0.62075 | 2.0721 | 0 | 0 |
| GO:0001533 | cornified envelope | 63 | 36 | 0.62711 | 2.0094 | 0 | 0 |
| GO:0031983 | vesicle lumen | 320 | 87 | 0.48934 | 1.8991 | 0 | 0.0022305 |
| GO:0005840 | ribosome | 218 | 93 | 0.44460 | 1.6700 | 0 | 0.030026 |
| GO:0015629 | actin cytoskeleton | 459 | 116 | 0.38444 | 1.5355 | 0 | 0.066605 |
| GO:0005798 | Golgi-associated vesicle | 161 | 36 | 0.41714 | 1.5056 | 0 | 0.079828 |
| GO:0031012 | extracellular matrix | 475 | 74 | 0.35691 | 1.4355 | 0 | 0.11279 |
| GO:1990351 | transporter complex | 317 | 115 | -0.48843 | -1.5918 | 0 | 0.15 |
| **GO: Molecular Function** | | | | | | | |
| GO:0003735 | structural constituent of ribosome | 154 | 66 | 0.58576 | 2.1312 | 0 | 0 |
| GO:0050839 | cell adhesion molecule binding | 449 | 104 | 0.52396 | 2.0571 | 0 | 0 |
| GO:0042393 | histone binding | 175 | 54 | -0.55193 | -1.7018 | 0 | 0.061919 |
| GO:0043021 | ribonucleoprotein complex binding | 117 | 37 | 0.42105 | 1.4389 | 0 | 0.12948 |
| GO:0003779 | actin binding | 399 | 103 | 0.37310 | 1.4658 | 0 | 0.13118 |
| GO:0060090 | molecular adaptor activity | 190 | 47 | 0.37758 | 1.4218 | 0 | 0.13338 |
| GO:0001882 | nucleoside binding | 357 | 87 | 0.36878 | 1.4412 | 0 | 0.13479 |
| GO:0001228 | DNA-binding transcription activator activity, RNA polymerase II-specific | 436 | 129 | -0.45530 | -1.5001 | 0 | 0.29583 |
| GO:0022803 | passive transmembrane transporter activity | 441 | 137 | -0.40413 | -1.3370 | 0 | 0.47921 |
| GO:0046873 | metal ion transmembrane transporter activity | 424 | 138 | -0.41574 | -1.3801 | 0 | 0.48601 |
| **KEGG Pathway** | | | | | | | |
| hsa03050 | Proteasome | 44 | 27 | 0.81882 | 2.4775 | 0 | 0 |
| hsa04510 | Focal adhesion | 194 | 40 | 0.57977 | 2.1909 | 0 | 0 |
| hsa03010 | Ribosome | 131 | 56 | 0.60652 | 2.1039 | 0 | 0 |
| hsa05204 | Chemical carcinogenesis | 75 | 32 | -0.64782 | -1.8411 | 0 | 0 |
| hsa04657 | IL-17 signaling pathway | 89 | 29 | 0.52112 | 1.7342 | 0 | 0.027467 |
| hsa05205 | Proteoglycans in cancer | 196 | 41 | 0.46039 | 1.7104 | 0 | 0.032176 |
| hsa04145 | Phagosome | 145 | 30 | 0.48266 | 1.6856 | 0 | 0.036008 |
| hsa04621 | NOD-like receptor signaling pathway | 164 | 46 | 0.43408 | 1.5918 | 0 | 0.060821 |
| hsa04144 | Endocytosis | 228 | 55 | 0.42629 | 1.5945 | 0 | 0.061759 |
| hsa03008 | Ribosome biogenesis in eukaryotes | 70 | 30 | 0.49625 | 1.5857 | 0 | 0.062312 |

**Suppl.Table 7.** PLEK2-related kinase targets and transcription factor targets (LinkedOmics).

| Enriched Category | Geneset | Leading Edge Number | NES | FDR |
| --- | --- | --- | --- | --- |
| Kinase Target | Kinase_PTK2 | 5 | 1.9097 | 0.040438 |
| Transcription Factor Target | V$SRF_01 | 9 | 1.9966 | 0 |
|  | V$CART1_01 | 64 | -1.7070 | 0.026054 |
|  | V$NRF2_01 | 58 | 1.5399 | 0.035499 |
|  | V$MYC_Q2 | 40 | 1.5025 | 0.038906 |
|  | V$AP1_01 | 60 | 1.4459 | 0.046938 |

**Suppl.Table 8.** The Spearman’s coefficients of the top 10 hub genes associated with PLEK2 obtained from LinkedOmics, CbioPortal and GEPIA2 database.

|  | LinkedOmics | | | CbioPortal | | | GEPIA2 | |
| --- | --- | --- | --- | --- | --- | --- | --- | --- |
|  | Correlation | P-value | FDR (BH) | Correlation | P-value | q-value | Correlation | P−value |
| ITGB4 | 0.574264924 | 5.94E-47 | 1.74E-44 | 0.568890961 | 7.12E-44 | 1.89E-41 | 0.66 | 5.8e−72 |
| ITGB1 | 0.479375294 | 3.1E-31 | 1.57E-29 | 0.471419131 | 8.28E-29 | 3.80E-27 | 0.47 | 6.2e−33 |
| ITGA6 | 0.52356615 | 6.13E-38 | 6.21E-36 | 0.526950461 | 8.66E-37 | 9.61E-35 | 0.55 | 4.3e−46 |
| ITGA5 | 0.458435637 | 2.20E-28 | 8.35E-27 | 0.461253739 | 1.69E-27 | 6.77E-26 | 0.52 | 4.5e−41 |
| ITGA3 | 0.728862317 | 2.87E-87 | 2.89E-83 | 0.724367417 | 8.00E-82 | 1.62E-77 | 0.76 | 3e−109 |
| LAMA3 | 0.634076447 | 7.89E-60 | 8.37E-57 | 0.629490629 | 4.21E-56 | 4.48E-53 | 0.74 | 3.2e−97 |
| LAMC2 | 0.616374737 | 9.95E-56 | 8.36E-53 | 0.619044275 | 8.41E-54 | 7.38E-51 | 0.67 | 2.8e−75 |
| LAMB3 | 0.535584588 | 6.13E-40 | 7.68E-38 | 0.542133544 | 3.07E-39 | 4.56E-37 | 0.6 | 1.4e−56 |
| PXN | 0.694929635 | 3.25E-76 | 1.31E-72 | 0.694829682 | 9.73E-73 | 4.91E-69 | 0.66 | 9.2e−72 |
| ITGB6 | 0.449263615 | 3.39E-27 | 1.14E-25 | 0.461684083 | 1.49E-27 | 6.01E-26 | 0.48 | 4.4e−33 |

**Suppl.Table 9.** Gene enrichment terms of the top 10 hub genes associated with PLEK2 using R package “clusterProfiler”.

| **ID** | | **Description** | **GeneRatio** | | **pvalue** | | **p.adjust** | | **qvalue** | |
| --- | --- | --- | --- | --- | --- | --- | --- | --- | --- | --- |
| **GO:Biological Process** | | | | | | | | | | |
| \| GO:0030198 \| extracellular matrix organization \| 9/10 \| 2.41E-15 \| 6.17E-13 \| 2.76E-13 \| \| --- \| --- \| --- \| --- \| --- \| --- \| \| GO:0031581 \| hemidesmosome assembly \| 5/10 \| 7.57E-15 \| 7.83E-13 \| 3.51E-13 \| \| GO:0043062 \| extracellular structure organization \| 9/10 \| 9.18E-15 \| 7.83E-13 \| 3.51E-13 \| \| GO:0007044 \| cell-substrate junction assembly \| 6/10 \| 4.17E-12 \| 2.67E-10 \| 1.20E-10 \| \| GO:0001704 \| formation of primary germ layer \| 6/10 \| 1.40E-11 \| 7.18E-10 \| 3.22E-10 \| \| GO:0007369 \| gastrulation \| 6/10 \| 1.80E-10 \| 7.67E-09 \| 3.44E-09 \| \| GO:0034329 \| cell junction assembly \| 6/10 \| 5.47E-10 \| 2.00E-08 \| 8.97E-09 \| \| GO:0034330 \| cell junction organization \| 6/10 \| 2.58E-09 \| 8.27E-08 \| 3.70E-08 \| \| GO:0031589 \| cell-substrate adhesion \| 6/10 \| 6.37E-09 \| 1.81E-07 \| 8.12E-08 \| \| GO:0007160 \| cell-matrix adhesion \| 5/10 \| 5.57E-08 \| 1.43E-06 \| 6.40E-07 \|   **GO: Cellular Component**   \| GO:0008305 \| integrin complex \| 5/10 \| 1.89E-12 \| 1.05E-10 \| 2.97E-11 \| \| \| --- \| --- \| --- \| --- \| --- \| --- \| --- \| \| GO:0098636 \| protein complex involved in cell adhesion \| 5/10 \| 3.14E-12 \| 1.05E-10 \| 2.97E-11 \| \| \| GO:0030055 \| cell-substrate junction \| 7/10 \| 2.39E-10 \| 5.33E-09 \| 1.51E-09 \| \| \| GO:0043235 \| receptor complex \| 6/10 \| 2.76E-09 \| 4.63E-08 \| 1.31E-08 \| \| \| GO:0098802 \| plasma membrane receptor complex \| 5/10 \| 8.42E-09 \| 1.13E-07 \| 3.19E-08 \| \| \| GO:0005925 \| focal adhesion \| 6/10 \| 1.74E-08 \| 1.71E-07 \| 4.84E-08 \| \| \| GO:0005924 \| cell-substrate adherens junction \| 6/10 \| 1.79E-08 \| 1.71E-07 \| 4.84E-08 \| \| \| GO:0009897 \| external side of plasma membrane \| 5/10 \| 3.15E-08 \| 2.64E-07 \| 7.46E-08 \| \| \| GO:0005912 \| adherens junction \| 6/10 \| 6.53E-08 \| 4.86E-07 \| 1.37E-07 \| \| GO:0005604 \| basement membrane \| 4/10 \| 1.08E-07 \| 7.25E-07 \| 2.05E-07 \|   **GO: Molecular Function**   \| GO:0050839 \| cell adhesion molecule binding \| 5/10 \| 2.71E-06 \| 9.49E-05 \| 3.42E-05 \| \| --- \| --- \| --- \| --- \| --- \| --- \| \| GO:0001618 \| virus receptor activity \| 3/10 \| 9.35E-06 \| 0.000109 \| 3.94E-05 \| \| GO:0104005 \| hijacked molecular function \| 3/10 \| 9.35E-06 \| 0.000109 \| 3.94E-05 \| \| GO:0031994 \| insulin-like growth factor I binding \| 2/10 \| 1.40E-05 \| 0.000123 \| 4.43E-05 \| \| GO:0005178 \| integrin binding \| 3/10 \| 3.34E-05 \| 0.000233 \| 8.43E-05 \| \| GO:0001968 \| fibronectin binding \| 2/10 \| 8.57E-05 \| 0.000392 \| 0.000142 \| \| GO:0005201 \| extracellular matrix structural constituent \| 3/10 \| 9.06E-05 \| 0.000392 \| 0.000142 \| \| GO:0005520 \| insulin-like growth factor binding \| 2/10 \| 0.000101 \| 0.000392 \| 0.000142 \| \| GO:0043236 \| laminin binding \| 2/10 \| 0.000101 \| 0.000392 \| 0.000142 \| \| GO:0050840 \| extracellular matrix binding \| 2/10 \| 0.000279 \| 0.000975 \| 0.000352 \| \| GO:0005518 \| collagen binding \| 2/10 \| 0.000562 \| 0.001787 \| 0.000645 \| \| GO:0002020 \| protease binding \| 2/10 \| 0.002186 \| 0.005885 \| 0.002124 \|   **KEGG Pathway** | | | | | | | | | | |
| hsa04512 | ECM-receptor interaction | | | 9/10 | | 1.69E-17 | | 5.59E-16 | | 2.32E-16 |
| hsa04510 | Focal adhesion | | | 10/10 | | 8.08E-17 | | 1.33E-15 | | 5.53E-16 |
| hsa05165 | Human papillomavirus infection | | | 10/10 | | 1.39E-14 | | 1.53E-13 | | 6.35E-14 |
| hsa04151 | PI3K-Akt signaling pathway | | | 9/10 | | 6.24E-12 | | 5.15E-11 | | 2.14E-11 |
| hsa05412 | Arrhythmogenic right ventricular cardiomyopathy (ARVC) | | | 6/10 | | 1.42E-10 | | 9.35E-10 | | 3.88E-10 |
| hsa05410 | Hypertrophic cardiomyopathy (HCM) | | | 6/10 | | 3.70E-10 | | 2.00E-09 | | 8.28E-10 |
| hsa05222 | Small cell lung cancer | | | 6/10 | | 4.24E-10 | | 2.00E-09 | | 8.28E-10 |
| hsa05414 | Dilated cardiomyopathy (DCM) | | | 6/10 | | 5.50E-10 | | 2.27E-09 | | 9.40E-10 |
| hsa04810 | Regulation of actin cytoskeleton | | | 7/10 | | 1.04E-09 | | 3.81E-09 | | 1.58E-09 |
| hsa05145 | Toxoplasmosis | | | 5/10 | | 1.24E-07 | | 4.08E-07 | | 1.69E-07 |


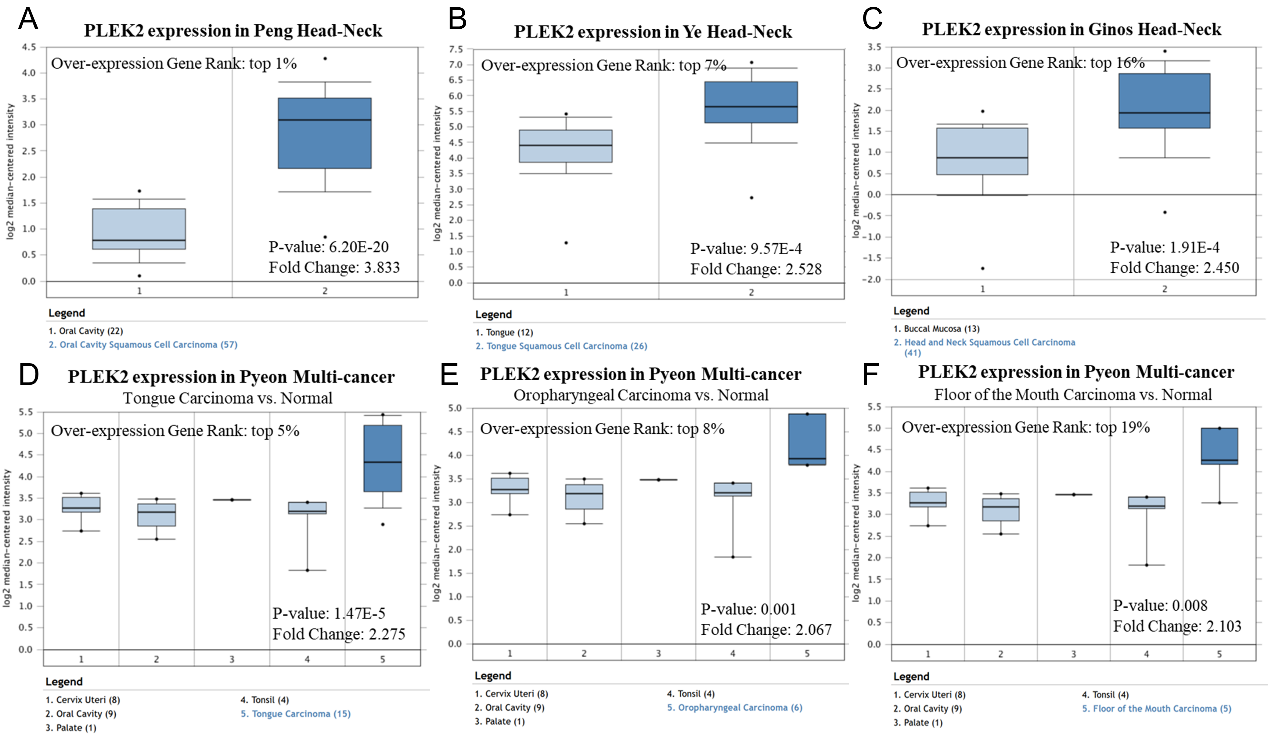


**Suppl. Figure 1.** Differential expression of PLEK2 in HNSCC using Oncomine database. (A) Peng-Head Neck, (B)Ye-Head Neck, (C) Ginos-Head Neck. (D) Pyeon Multi-cancer (Tongue Carcinoma). (E) Pyeon Multi-cancer (Oropharyngeal Carcinoma). (F) Pyeon Multi-cancer (Floor of the Mouth Carcinoma).


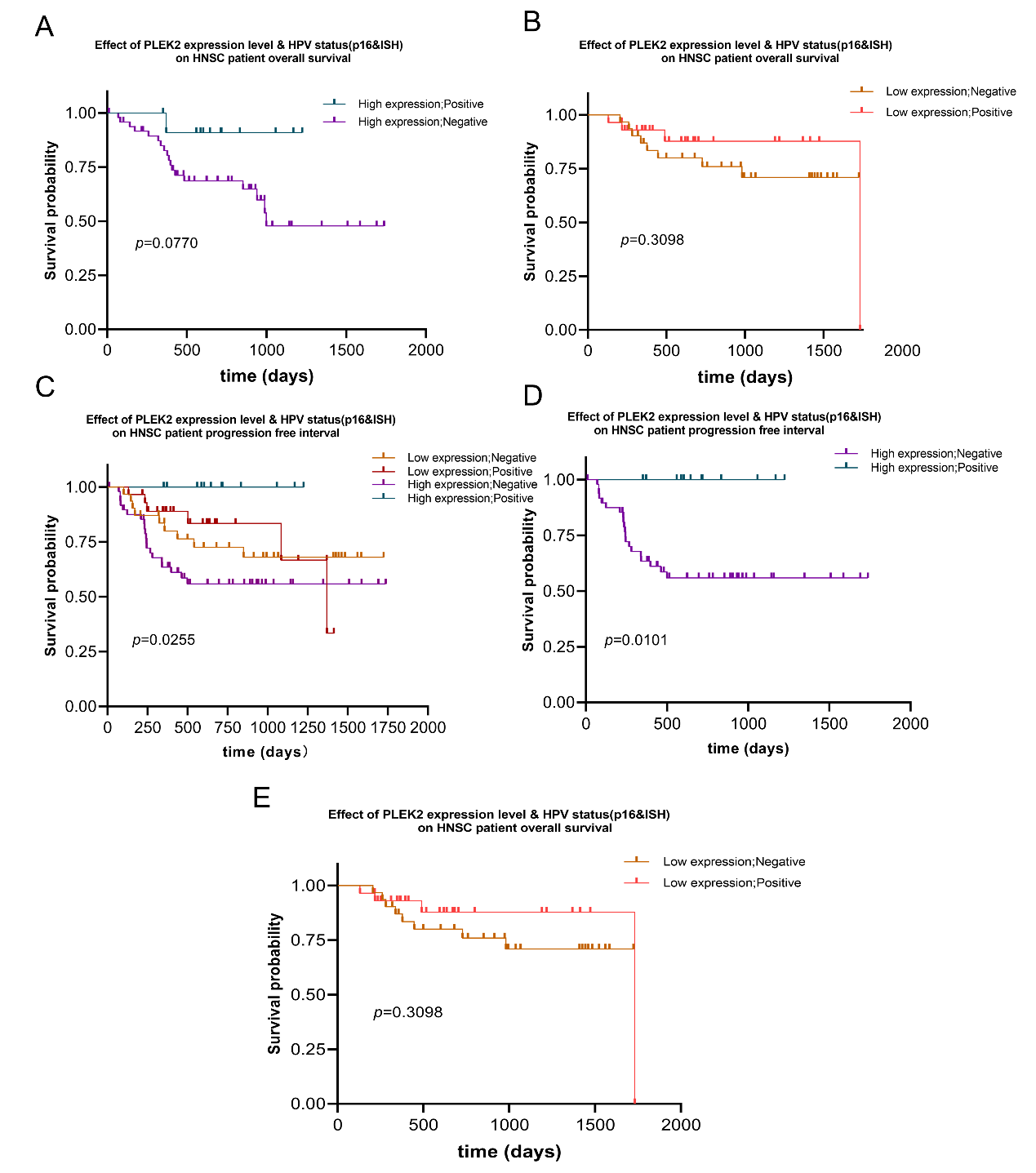


**Suppl. Figure 2.** Subgroup survival analysis of HNSCC patients using UCSC Xena browser based on HPV status. (A) Overall survival comparisons between PLEK2 high expression & HPV+ and PLEK2 high expression & HPV- subgroups. (B) PLEK2 low expression & HPV+ and PLEK2 low expression & HPV- subgroups. (C) Progression-free interval comparisons among PLEK2 high expression & HPV+, PLEK2 high expression & HPV-, PLEK2 low expression & HPV+, and PLEK2 low expression & HPV- subgroups. (D) PLEK2 high expression & HPV+ and PLEK2 high expression & HPV- subgroups. (E) PLEK2 low expression & HPV+ and PLEK2 low expression & HPV- subgroups.


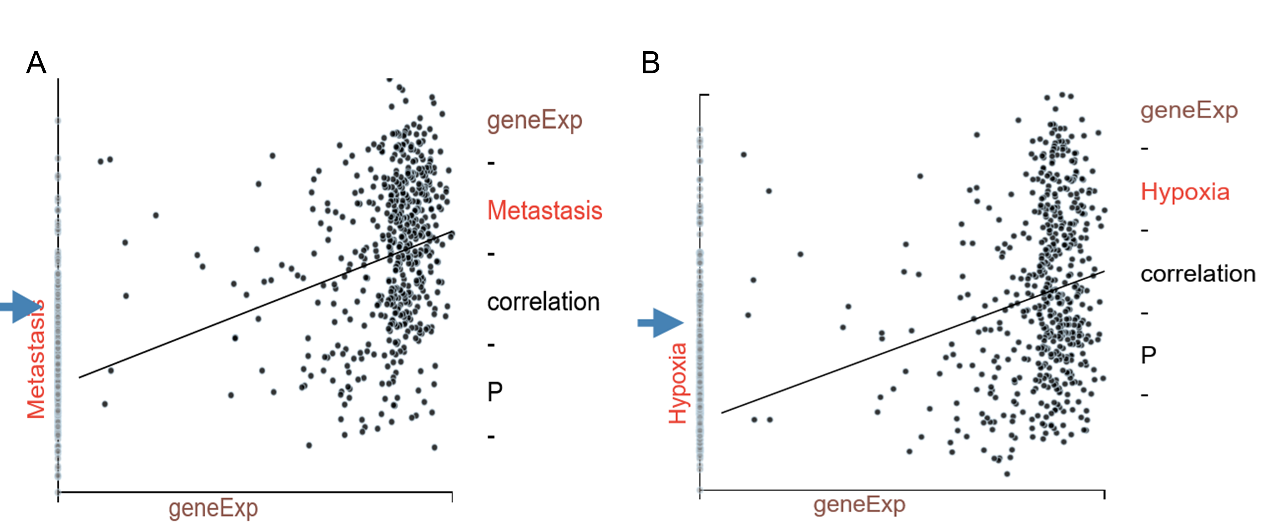


**Suppl. Figure 3.** The scatter plots indicating the relationship between PLEK2 expression and functional states. (A) metastasis. (B) Hypoxia.


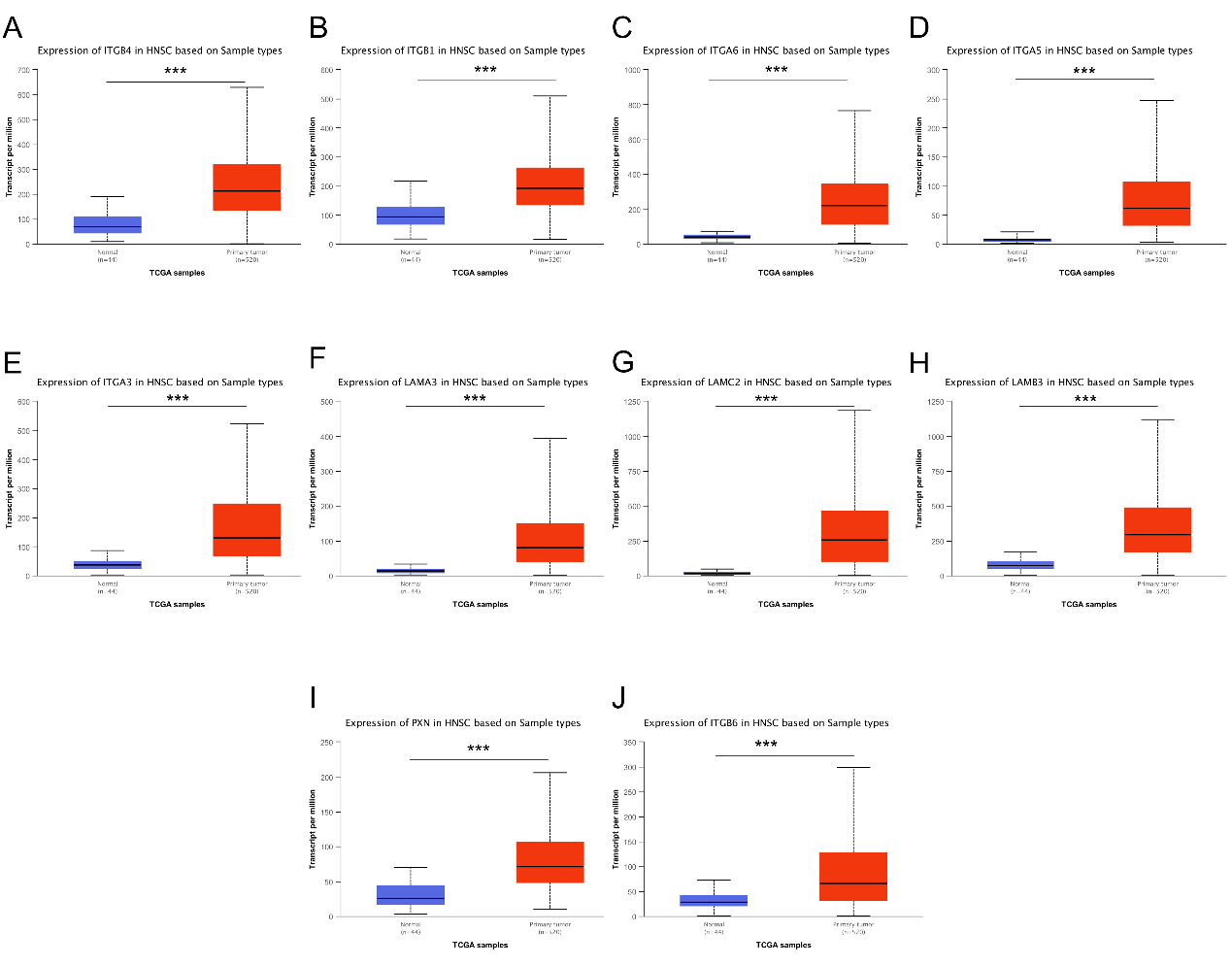


**Suppl. Figure 4.** Expression analysis of the top 10 hub genes by UALCAN database. (A) ITGB4. (B) ITGB1. (C) ITGA6. (D) ITGA5. (E) ITGA3. (F) LAMA3. (G) LAMC2. (H) LAMB3. (I) PXN. (J) ITGB6.


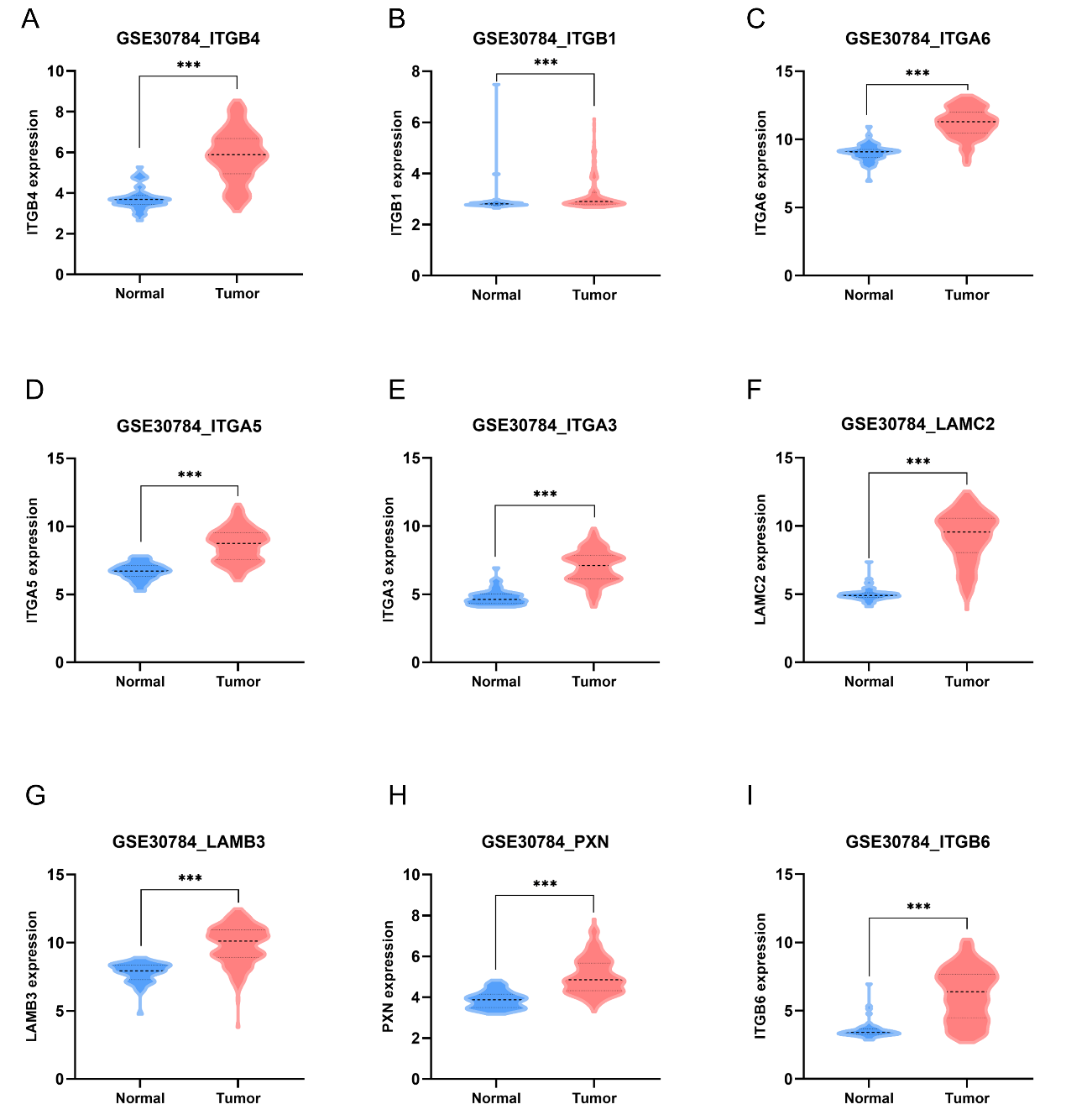


**Suppl. Figure 5.** Expression analysis of the 9 hub genes by GEO dataset (GSE30784). (A) ITGB4. (B) ITGB1. (C) ITGA6. (D) ITGA5. (E) ITGA3. (F) LAMC2. (G) LAMB3. (H) PXN. (I) ITGB6.


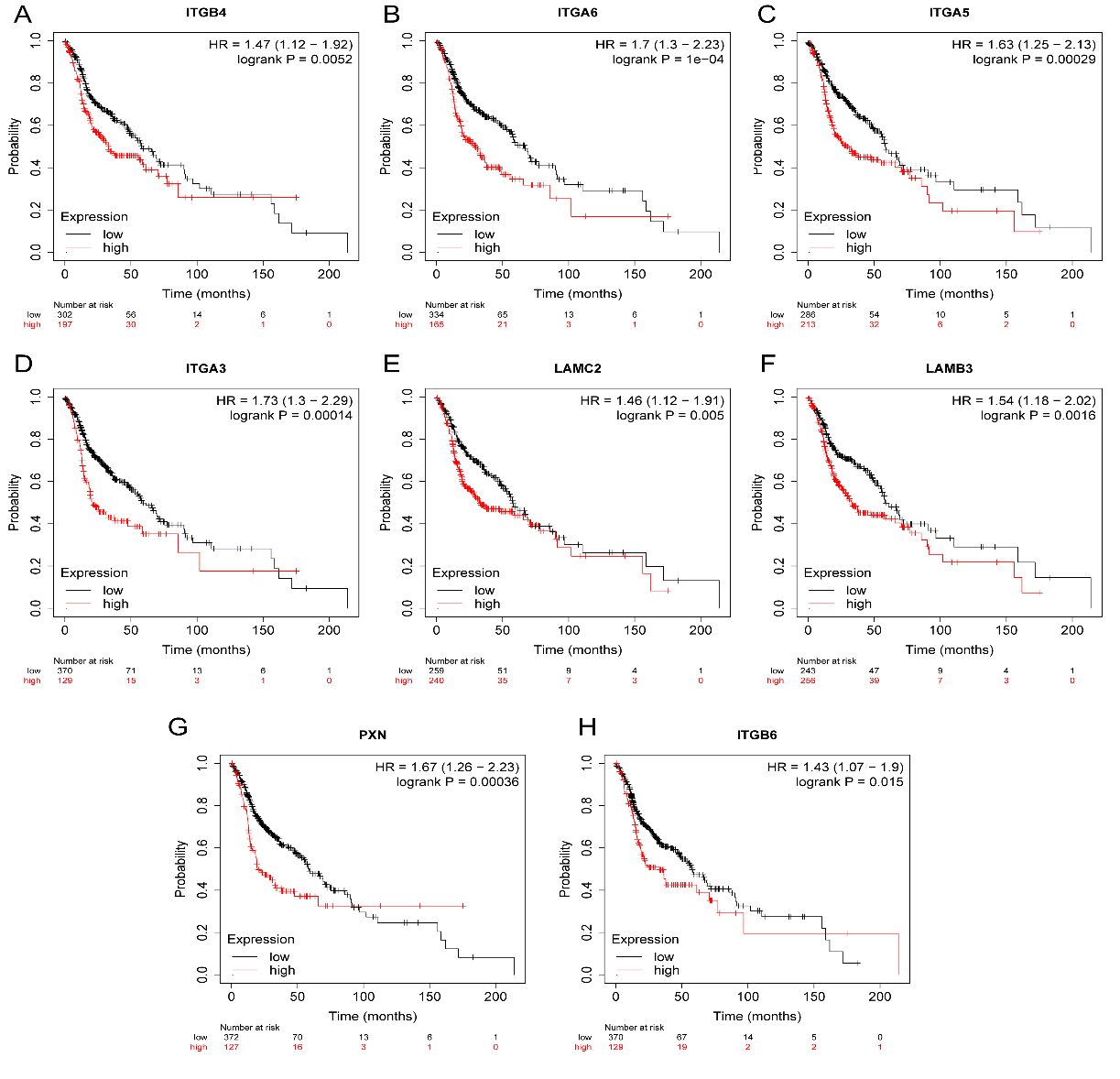


**Suppl. Figure 6.** Prognosis analysis of the 8 hub genes by Kaplan-Meier plotter database. (A) ITGB4. (B) ITGA6. (C) ITGA5. (D) ITGA3. (E) LAMC2. (F) LAMB3. (G) PXN. (H) ITGB6.


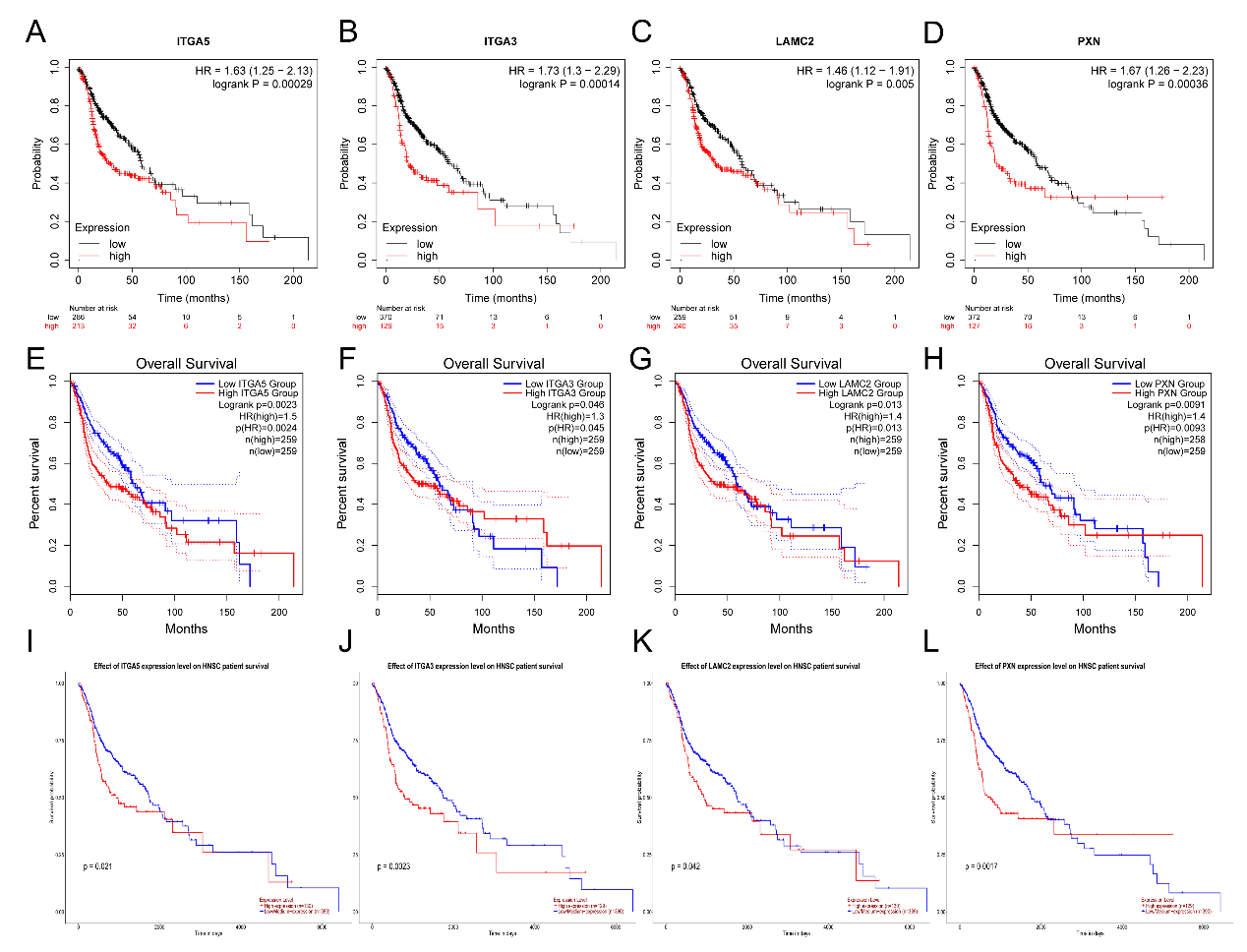


**Suppl. Figure 7.** Prognosis analysis of the 4 hub genes by (A-D) Kaplan-Meier plotter database, (E-H) GEPIA2 database, (I-L) UALCAN database. (A, E, I) ITGA5. (B, F, J) ITGA3. (C, G, K) LAMC2. (D, H, L) PXN.
